# Sodium–Glucose Cotransporter 2 Inhibitors in Diabetic Solid Organ Transplant Recipients: A Systematic Review and Meta‐Analysis of Comparative Studies

**DOI:** 10.1155/jdr/8540354

**Published:** 2026-01-19

**Authors:** Min Jung Geum, Kyung Sun Oh, Young-Mi Ah

**Affiliations:** ^1^ Department of Pharmacy, Severance Hospital, Yonsei University Health System, Seoul, Republic of Korea, yuhs.or.kr; ^2^ College of Pharmacy, Yeungnam University, Gyeongsan, Republic of Korea, yu.ac.kr

## Abstract

**Background:**

Solid organ transplant recipients with diabetes mellitus face unique challenges in glycemic control, compounded by the metabolic effects of immunosuppressants. Although sodium–glucose cotransporter 2 (SGLT2) inhibitors are effective in diabetes, evidence for their use in transplant recipients remains limited. We aimed to systematically review the efficacy and safety of SGLT2 inhibitors in transplant recipients with diabetes.

**Methods:**

A systematic review and meta‐analysis was conducted by searching the MEDLINE, Embase, and Cochrane CENTRAL databases for studies published before July 2025. Seventeen comparative studies involving 12,892 transplant recipients with diabetes were included. Efficacy and safety data of SGLT2 inhibitors were extracted and analyzed. A random‐effects model was used to pool the results.

**Results:**

SGLT2 inhibitors led to a significantly greater reduction in HbA1c (mean difference [MD]: −0.59% and 95% confidence interval [CI]: −0.91 to −0.26) and body mass index (MD: −0.82 kg/m^2^ and 95% CI: −1.54 to −0.10) compared to controls. The risks of dialysis (odds ratio [OR]: 0.50 and 95% CI: 0.31–0.79), major adverse cardiovascular events (OR: 0.29 and 95% CI: 0.22–0.38), heart failure (OR: 0.66 and 95% CI: 0.52–0.83), urinary tract infection (OR: 0.45 and 95% CI: 0.22–0.92), graft rejection (OR: 0.73, 95% and CI: 0.64–0.83), and all‐cause mortality (OR: 0.40 and 95% CI: 0.27–0.59) were significantly lower in the SGLT2 inhibitor group.

**Conclusions:**

SGLT2 inhibitors were associated with improved glycemic control, weight reduction, and favorable trends in cardiovascular outcomes among solid organ transplant recipients. Most safety outcomes, including urinary tract infection and graft rejection, were comparable between groups or more favorable in the SGLT2 inhibitor group, particularly among kidney transplant recipients. These findings support the use of SGLT2 inhibitors in this population. However, further large‐scale studies are warranted to validate these results and assess the long‐term effects across different transplant types.

## 1. Introduction

Solid organ transplantation has been steadily increasing and is accompanied by significant improvements in clinical outcomes. The number of transplants performed increased by approximately 70% from 2001 to 2021 [1]. Additionally, the median survival rates for most solid organ transplants have improved [2]. With overall survival increasing, long‐term complication management in solid organ recipients has become a critical aspect of patient care.

Diabetes mellitus is a complication of the use of immunosuppressive agents in solid organ transplant recipients [3]. A Danish cohort study reported that the incidence of posttransplantation diabetes mellitus (PTDM) between Days 46 and 365 after transplantation was 3.80 per 100 person‐years of follow‐up [4]. Considering that end‐stage renal disease caused by diabetic complications is a leading indication for kidney transplantation and that diabetes is highly prevalent among patients with liver cirrhosis [5, 6], the overall prevalence of diabetes is expected to be higher in solid organ transplant recipients than in the general population. Glycemic control in solid organ transplant recipients is often challenging because of the adverse metabolic effects of immunosuppressive agents [3]. These factors underscore the significant impact of diabetes and its management in this population.

Sodium–glucose cotransporter 2 (SGLT2) inhibitors have emerged as essential agents for diabetes treatment since 2012 [7]. Clinical guidelines primarily recommend them as first‐line antidiabetic agents for patients with cardiovascular disease, heart failure (HF), and/or chronic kidney disease (CKD) [8]. Nonetheless, evidence on the efficacy of SGLT2 inhibitors in transplant recipients is limited. PTDM often arises as a consequence of immunosuppressive therapy, such as corticosteroids and calcineurin inhibitors, which impair insulin secretion and increase insulin resistance. This distinct pathophysiology differentiates PTDM from Type 2 diabetes in the general population, highlighting the need for dedicated studies evaluating the efficacy and safety of SGLT2 inhibitors in transplant recipients. Additionally, the use of SGLT2 inhibitors has been associated with an increased urogenital infection risk; genital infections occurred in 2.24% of users compared with 0.4% in the placebo group [9], highlighting the need for caution. Because infections are a major complication in transplant recipients due to their immunosuppressed state, ensuring the safety of SGLT2 inhibitors in this population is of particular importance. In this context, the Kidney Disease: Improving Global Outcomes (KDIGO) guidelines previously did not recommend SGLT2 inhibitors for kidney transplant recipients owing to insufficient evidence [10]; although recent updates have broadened the recommendations to adults with CKD [11], robust clinical data in transplant populations remain limited.

Several studies have evaluated the efficacy and safety of SGLT2 inhibitors in transplant recipients using real‐world data. Nevertheless, most previous systematic reviews or meta‐analyses have focused on kidney transplantation and on single‐arm before‐and‐after changes, as the available studies were primarily case series or lacked control groups [12–15]. To appropriately evaluate the efficacy and safety of SGLT2 inhibitors in solid organ transplant recipients, comparative analyses with control groups are necessary; however, to date, no meta‐analysis has incorporated such comparisons.

Therefore, we aimed to systematically review the efficacy and safety of SGLT2 inhibitors in transplant recipients with diabetes, incorporate recent literature, and quantitatively analyze their efficacy and safety based on comparative studies. Moreover, given the heterogeneity of immunosuppressive regimens and metabolic profiles among transplant populations, we aimed to assess the efficacy and safety of SGLT2 inhibitors across various types of solid organ transplantation.

## 2. Materials and Methods

This study followed the Preferred Reporting Items for Systematic Reviews and Meta‐Analyses (PRISMA) 2020 guidelines (Table S1) [16]. The study protocol is available on the PROSPERO database (CRD42024627940). Two investigators (M.J.G. and K.S.O.) independently performed a literature search, study selection, data extraction, and quality assessment. Any discrepancies were resolved through discussions with Y‐M.A.

### 2.1. Search Strategy

The MEDLINE, Embase, and Cochrane CENTRAL electronic databases were systematically searched for relevant studies published before July 2025 using a combination of medical subject headings and the keywords “transplant” and “SGLT2 inhibitors.” The complete search strategy is presented in Table S2.

### 2.2. Study Selection

Studies were considered eligible for inclusion if they met the following criteria: (i) involved solid organ transplant recipients with preexisting diabetes mellitus or PTDM; (ii) evaluated the efficacy and/or safety of SGLT2 inhibitors, either as a primary research objective or as part of broader comparative analyses with other antidiabetic agents, using clinical outcomes; and (iii) were comparative studies. The following studies were excluded: (i) nonhuman studies; (ii) reviews, meta‐analyses, and ongoing studies; (iii) case series or case reports; (iv) studies available only in the form of abstracts or posters; (v) studies evaluating combinations of SGLT2 inhibitors and other antidiabetic medications; and (vi) studies published in languages other than English.

### 2.3. Data Extraction and Outcomes

Eligible studies were reviewed, and the following data were extracted using a standardized extraction form: first author, publication year, country, study design, number of patients, sex, age, body mass index (BMI), estimated glomerular filtration rate (eGFR), hemoglobin A1c (HbA1c), type of SGLT2 inhibitors, comparators, transplantation history, diabetes mellitus history, comorbidities, immunosuppressive therapy including drug concentration level, other concomitant medication used, and follow‐up duration. The efficacy outcomes included HbA1c, BMI, body weight, eGFR, serum creatinine (SCr), dialysis, proteinuria, systolic blood pressure (SBP), diastolic blood pressure (DBP), and cardiovascular events, including major adverse cardiovascular events (MACEs), HF, and myocardial infarction (MI). Safety outcomes included the incidence of urinary tract infection (UTI), genital mycotic infection, acute kidney injury (AKI), diabetic (or euglycemic) ketoacidosis, graft rejection, hospitalization, mortality, and discontinuation of SGLT2 inhibitors.

### 2.4. Analyses

We conducted meta‐analyses of the outcomes by including all studies initially identified. For continuous outcomes, we reported mean differences (MDs) with 95% confidence intervals (CIs) calculated using the inverse variance method. The MDs were obtained as the difference in the mean changes from baseline to follow‐up between the intervention and control groups. For studies with follow‐up of 12 months or longer that reported multiple time points, 12‐month outcomes were used; otherwise, outcomes were used according to the follow‐up period. When the mean changes were not reported, they were derived from the available data. If only the median and interquartile range were reported, means and SDs were estimated using the method proposed by Wan et al. [17, 18]. Graphically reported values were extracted using WebPlotDigitizer Version 5.2 [19]. The missing SDs were imputed using the method described in the *Cochrane Handbook for Systematic Reviews of Interventions*, Version 6.5 [18]. For binary outcomes, pooled odds ratios (ORs) with 95% CIs were calculated using the Mantel–Haenszel methods, whereas pooled adjusted hazard ratios (HRs) were estimated using the inverse variance method. Studies with zero events in both groups were excluded from the meta‐analysis. In addition, outcomes were not included in the meta‐analysis if they were reported in noncombinable units (e.g., proteinuria) or were available from only a single study. Heterogeneity was assessed using the *I* ^2^ statistic, with the desired threshold set at *I* ^2^ > 50*%* [20]. A random‐effects model was used because of the expected clinical heterogeneity in the included populations [18]. Subgroup analyses were conducted separately in kidney and heart transplant recipients. Sensitivity analysis was performed by removing one study per analysis (leave‐one‐out), and additional analyses were conducted by including only randomized controlled trials and observational studies with moderate or higher methodological quality, as determined by our risk of bias assessment. In addition to a meta‐analysis comparing the efficacy and safety of SGLT2 inhibitors with those of a control group, an analysis was performed to evaluate the efficacy and safety of SGLT2 inhibitors before and after drug administration. In the single‐arm analysis, all studies were included regardless of the risk of bias.

The quality of each included study was assessed using the Risk of Bias in Nonrandomized Studies of Interventions tool for nonrandomized comparative studies [21] and the Risk of Bias 2 tool for a randomized controlled trial (RCT) [22]. For outcomes with at least 10 included studies, publication bias was assessed using Egger′s regression test and visual inspection of funnel plots for asymmetry [23]. When asymmetry was detected, the trim‐and‐fill method was applied to explore the potential impact of missing studies. Statistical significance was set at *p* < 0.05. All statistical analyses were performed using R (Version 4.2.1).

## 3. Results

### 3.1. Study Selection

Figure S1 presents the study selection process according to the PRISMA 2020 guidelines. After removing duplicates, 722 articles were screened by title and abstract, and 621 articles were excluded. The remaining 101 articles underwent full‐text assessment, resulting in 17 comparative studies involving 12,892 patients.

### 3.2. Study Characteristics

Table 1 summarizes the characteristics of the 17 studies. One was a double‐blind placebo‐controlled RCT, whereas the others were retrospective cohort studies [24]. There were ten studies on kidney, six on heart, and one on liver transplant recipients (Table 1). The number of participants ranged from 37 to 3,940 per study. The mean or median age of the participants ranged from 52.1 to 65.0 years. Most studies had a 12‐month follow‐up period, whereas others ranged from 3 months to over 6.9 years.

**Table 1:** Baseline characteristics of studies included.

**Author year (country)**	**Study design**	**Study groups**	**Sample** **size**	**Insulin,** **%**	**GLP-1 RAs,** **%**	**Other oral hypoglycemic agents, %**				**Age,** **years,** **mean**	**Male,** **%**	**HbA1c** **%**	**BMI** **kg/m** ^**2**^, **mean**	**eGFR,** **mL/min/1.73m** ^**2**^,	**Follow-up** **duration,** **months**
						**Metformin**	**DPP-4 inhibitors**	**Sulfonylureas**	**Meglitinides**						
Kidney transplantation															
Halden 2019 (Norway) [24]	Randomized controlled, double‐blind (single‐center)	*I*: E	22	22.7	—	4.6	36.4	13.6	—	63^a^	77.3	6.9^a^	28.8^a^	66^a^	6
		*C*: placebo	22	13.6	—	4.6	50.0	18.2	—	59^a^	77.3	6.8^a^	27.5^a^	59^a^	
Hidasome 2021 (Japan) [25]	Retrospective cohort (single‐center)	*I*: D, E, C, I, L, T	28	60.7	—	—	—	—	—	54.8	78.6	7.7	23.6	50.4	12
		*C*: non‐SGLT2I	57	76.1	—	—	—	—	—	55.7	75.4	7.6	23.4	47.5	
Lim 2022 (South Korea) [26]	Retrospective cohort (multicenter)	*I*: E, D	226	61.9 ^∗^	—	87.6 ^∗^	52.2	45.6 ^∗^	—	51.2	69.5	—	25.1	—	12 (SCr, eGFR, HbA1c), 62.9^b^
		*C*: non‐SGLT2I	1,857	54.7 ^∗^	—	55.3 ^∗^	55.3	33.5 ^∗^	—	52.6	66.9	—	23.7	—	
Demir 2023 (Turkey) [27]	Retrospective cohort (multicenter)	*I*: E, D	36	—	—	—	—	—	—	49.1 ^∗^	66.7	9.1 ^∗^	28.4	71.9	12
		*C*: non‐SGLT2I	21	—	—	—	—	—	—	55.0 ^∗^	57.1	6.4 ^∗^	28.6	73.2	
Mahmoud 2023 (Kuwait) [28]	Retrospective cohort (single‐center)	*I*: C	98	66.3	0	78.6	27.6 ^∗^	8.2	—	56	67.3	8.0^a^^∗^	31.4^a^	67.2^a^	12
		*C*: non‐SGLT2I and non GLP‐1 RA	70	52.9	0	84.3	55.7 ^∗^	11.4	—	57	58.6	7.2^a^^∗^	29.7^a^	63.8^a^	
Yeggalam 2023 (United States) [29]	Retrospective cohort (single‐center)	*I*: SGLT2I	44	77.3 ^∗^	31.8	29.5	—	—	—	61.7	65.9	7.9	30.9	55.9	12
		*C*: non‐SGLT2I	70	55.7 ^∗^	14.3	17.1	—	—	—	61.4	61.4	7.3	31.8	52.6	
Lim 2024 (South Korea) [30]	Retrospective cohort (multicenter)	*I*: SGLT2I	127	59.8	—	86.6 ^∗^	44.9 ^∗^	42.5	—	54.0^a^	68.5	—	25.3^a^	—	12 (SCr, HbA1c), 56.3^a^
		*C*: non‐SGLT2I	127	57.5	—	70.1 ^∗^	63.0 ^∗^	37.8	—	55.0^a^	72.4	—	25.2^a^	—	
Sheu 2024 (Taiwan) [31]	Retrospective cohort (multicenter)^i^	*I:* SGLT2I *C:* non‐SGLT2I	1,970 1,970	66.5 69.1	14.5 15.0	22.8 22.1	16.9 16.8	14.0 14.0	‐ ‐	59.5 59.6	63.2 63.2	7.27 7.23	29.7 29.9	54.0 53.5	40.8^a^
Diker Cohen 2025 (Israel) [32]	Retrospective cohort (single‐center)	*I:* E, D *C:* non‐SGLT2I	240 240	64.6 61.3	30.8 ^∗^ 8.8 ^∗^	58.3 ^∗^ 30.0 ^∗^	28.7 ^∗^ 20.8 ^∗^	5.8 2.9	14.6 17.5	64.0 63.0	80.0 80.0	7.7 ^∗^ 6.8 ^∗^	28.4 27.8	‐ ‐	36^a^ 27^a^
Yen 2025a (Taiwan) [33]	Retrospective cohort (multicenter)^i^	*I:* SGLT2I *C:* DPP‐4 inhibitors	1,410 1,410	46.6 44.6	4.6 4.1	14.5 14.5	0 1,410	10.5 10.1	—	61.4 61.4	60.1 59.5	N/A^g^	30.5 30.3	N/A^g^	≥12

Heart transplantation															
Muir 2017 (Australia) [34]	Retrospective cohort (single‐center)	*I*: E	16	31.3	0	68.8 ^∗^	12.5	31.3	—	55.0	81.3	7.3	29.8	57	≥3
		*C*: non‐SGLT2I	74	50.0	1.4	36.5 ^∗^	6.8	12.2	—	58.2	71.6	6.8	27.8	54	
Cehic 2019 (Australia) [35]	Retrospective cohort (single‐center)	*I*: E	22	40.9	—	68.2 ^∗^	13.6	27.3	—	59.3	77.3	7.5^a,c^	30.3^a^	48^a,c^	12
		*C*: non‐SGLT2I	79	54.4	—	22.8 ^∗^	7.6	12.5	—	58.0	68.4	6.9^a,d^	27.2^a^	54^a,d^	
Marfella 2022 (Italy) [36]	Prospective cohort (single‐center)	*I*: E, D, C	17	17.6	11.8	64.7	29.4	11.8	17.6	51.3	76.4	6.54	26.4	—	12
		*C*: non‐SGLT2I	20^e^	20.0	15.0	60.0	35.0	15.0	20.0	52.7	85.0	6.61	26.6	—	
Lu 2025 (Taiwan) [37]	Retrospective cohort (single‐center)	*I*: E, D *C:* non‐SGLT2I	50 35	36.0 37.1	—	62.0 40.0	80.0 100.0	10.0 20.0	28.0 14.3	56.6 61.4	92.0 ^∗^ 68.6 ^∗^	7.5 7.5	—	59.3 54.0	≥12
Raven 2025 (Australia) [38]	Retrospective cohort (single‐center)	*I*: SGLT2I *C:* non‐SGLT2I	23 81	—	—	—	—	—	—	58^a^ 54^a^	87.0 69.0	—	—	61.0^a^ 73.0^a^	≥36
Yen 2025b (Taiwan) [39]	Retrospective cohort (multicenter)^i^	*I*: SGLT2I *C:* non‐SGLT2I	1,063 1,063	58.6 57.3	8.6 8.2	27.6 30.4	10.3 12.4	12.0 14.0	—	58.8 59.7	70.4 72.0	N/A^h^	N/A^h^	N/A^h^	≥12

Liver transplantation															
Zheng 2024 (Canada) [40]	Retrospective cohort (single‐center)	*I*: E, D, C	87	88.5	0	37.9	—	—	—	65.0^a^	79.3	7.8^a^	28.1^a^	53.0^a^	12
		*C*: DPP‐4 inhibitors	217^f^	87.1	0	43.8	100	—	—	64.0^a^	79.7	6.9^a^	26.1^a^	56.0^a^	

Abbreviations: BMI, body mass index; C, canagliflozin; *C*, control group; D, dapagliflozin; DPP‐4 inhibitors, dipeptidyl peptidase‐4 inhibitors; E, empagliflozin; eGFR, estimated glomerular filtration rate; GLP‐1 RA, glucagon‐like peptide‐1 receptor agonist; HbA1c, hemoglobin A1c; *I*, intervention group; I, ipragliflozin; L, Luseogliflozin; SGLT2I, sodium–glucose cotransporter 2 inhibitors; SCr, serum creatinine; T, tofogliflozin.

^a^Median.

^b^Mean.

^c^In 20 patients of the intervention group followed for 12 months.

^d^In 77 patients of the control group followed for 12 months.

^e^Only diabetic patients; excluded nondiabetic patients.

^f^Only SGLT2i and comparator, excluded GLP1‐RA and combination therapy.

^g^Patients with HbA1c ≥ 7%: SGLT2I 44.6% vs. non‐SGLT2I 42.3% and patients with eGFR <60 mL/min/1.73m^2^: SGLT2I 63.8% vs. non‐SGLT2I 63.9%.

^h^Patients with HbAlc ≥7%: SGLT2I 45.4% vs. non‐SGLT2I 45.9%; patients with BMI ≥30: SGLT2I 42.9% vs. non‐SGLT2I 43.6%; patients with eGFR <60 mL/min/1.73m^2^: SGLT2I 66.1% vs. non‐SGLT2I 65.0%.

^i^Data from TriNetX database, a global collaborative health research platform.

^∗^Indicates a significant difference between the intervention group and control group.

Four studies evaluated a single SGLT2 inhibitor: empagliflozin in three [24, 34, 35] and canagliflozin in one [28]. Other studies investigated two or more SGLT2 inhibitors, whereas six studies did not specify the type of drug used [29, 31, 33, 38, 39]. Comparators across all studies were non‐SGLT2 inhibitors; one used nonglucagon‐like peptide‐1 receptor agonists [28], and another used dipeptidyl peptidase‐4 (DPP‐4) inhibitors [33, 40]. All studies allowed the concomitant use of other hypoglycemic agents, with insulin and metformin being the most commonly used.

Table S3 summarizes the disease characteristics and immunosuppressant use. Immunosuppressive regimens primarily included tacrolimus, mycophenolate, and corticosteroids, with no significant differences in corticosteroid usage rates between the intervention and control group (33.3%–100%). Details of the study design and outcomes are summarized in Table S4.

### 3.3. Risk of Bias Assessments

The RCT was assessed as having some concerns (Table S5). Four of the 16 nonrandomized studies exhibited a serious risk of bias due to missing descriptions of the confounding control methods. The other 12 studies had a moderate risk of bias due to selection bias and other issues (Figure S2).

### 3.4. Efficacy and Safety of SGLT2 Inhibitors in Comparison With the Control Groups

#### 3.4.1. Main Analysis

HbA1c levels were reported in 11 studies. HbA1c levels significantly decreased more in the SGLT2 inhibitor group compared with the control group (MD: −0.59% and 95% CI: −0.91 to −0.26) (Figure 1a). Furthermore, the SGLT2 inhibitor group exhibited a significantly greater reduction in BMI and body weight compared to the control group (MD: −0.82 kg/m^2^ and 95% CI: −1.54 to −0.10 (Figure 1b) MD: −3.0 kg and 95% CI: −4.08 to −1.92 (Table S6), respectively). Kidney function was primarily assessed using eGFR, and the meta‐analysis revealed no significant difference in eGFR changes between the SGLT2 inhibitor and control groups (MD: 2.67 mL/min/1.73 m^2^and 95% CI: −0.29 to 5.64) (Figure 1c). Despite the inclusion of only two studies, the risk of dialysis was significantly lower in the SGLT2 inhibitor group than in the control group (OR: 0.50 and 95% CI: 0.31–0.79) (Figure 1d). Additionally, the change in SCr level did not differ between the two groups (Table S6).

Figure 1Forest plot of efficacy outcomes. HbA1c, hemoglobin A1c; SGLT2I, sodium‐glucose cotransporter‐2 inhibitor; SD, standard deviation; MD, mean difference; CI, confidence interval; eGFR, estimated glomerular filtration rate; and OR, odds ratio.

**Figure:**
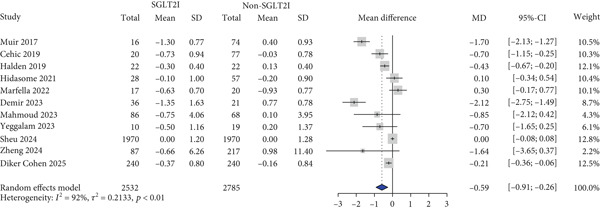
(a) HbA1c (%)

**Figure:**
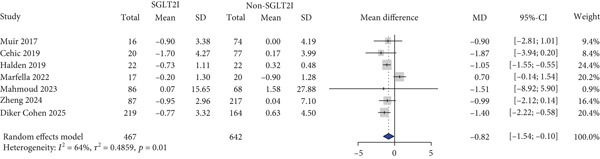
(b) Body mass index (kg/m2)

**Figure:**
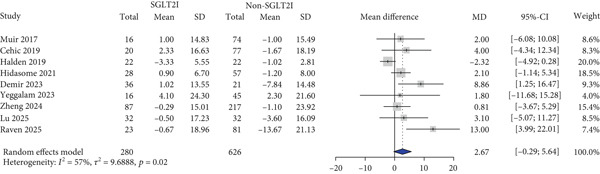
(c) eGFR (ml/min/1.73m2)

**Figure:**
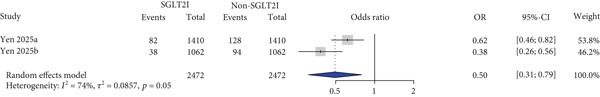
(d) Dialysis

**Figure:**
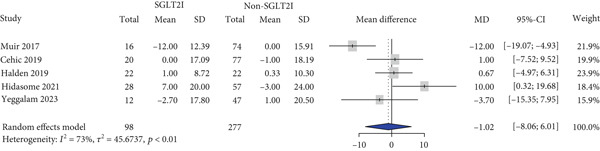
(e) Systolic blood pressure

**Figure:**
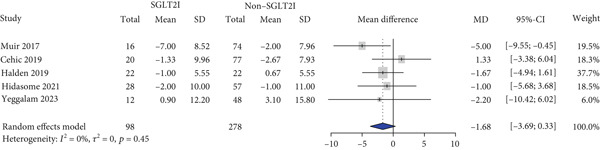
(f) Diastolic blood pressure

**Figure:**
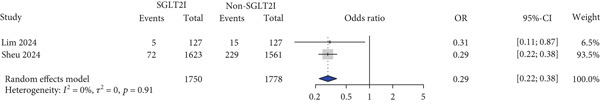
(g) Major adverse cardiovascular events

**Figure:**
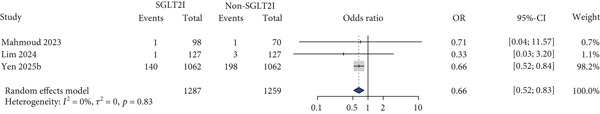
(h) Heart failure

**Figure:**
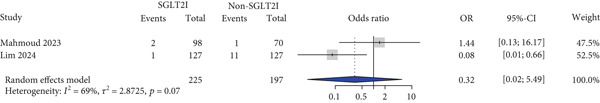
(i) Myocardial infarction

Blood pressure was evaluated in five studies, and there were no significant differences in blood pressure change between the two groups (SBP: MD, −1.02 mmHg and 95% CI, −8.06 to 6.01 and DBP: MD, −1.68 mmHg and 95% CI, −3.69 to 0.33) (Figure 1e,f). Cardiovascular events were reported in four studies. MACE (OR: 0.29 and 95% CI: 0.22–0.38) and HF (OR: 0.66 and 95% CI: 0.52–0.83) risks were lower in the SGLT2 inhibitor group than in the control group, whereas no significant difference was observed between the two groups regarding MI risk (OR: 0.32 and 95% CI: 0.02–5.49) (Figures 1g, 1h, and 1i).

Among infection‐related safety outcomes, UTI incidence was reported in 10 studies, with a significantly lower incidence in the SGLT2 inhibitor group compared with the control group (OR: 0.45 and 95% CI: 0.22–0.92) (Figure 2a), whereas the incidence of genital mycotic infection did not differ significantly between groups (Figure 2b).

Figure 2Forest plot of safety outcomes. OR, odds ratio; CI, confidence interval.

**Figure:**
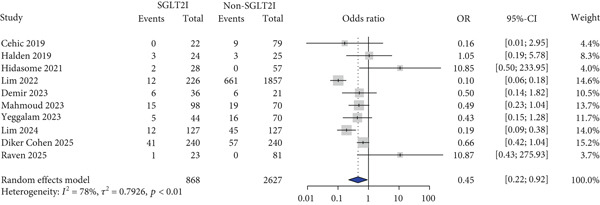
(a) Urinary tract infection

**Figure:**
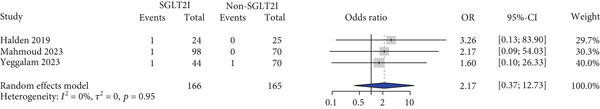
(b) Genital mycotic infection

**Figure:**
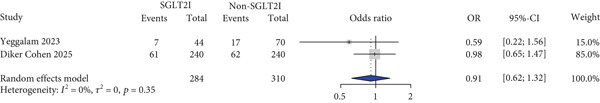
(c) Acute kidney injury

**Figure:**
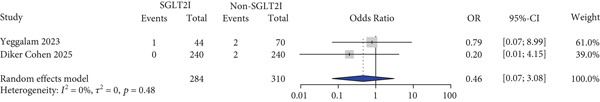
(d) Diabetic ketoacidosis

**Figure:**
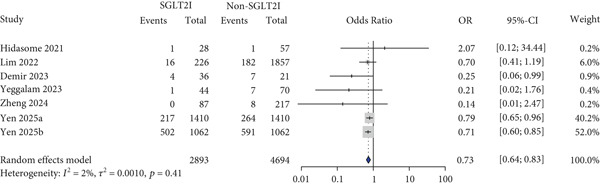
(e) Graft rejection

**Figure:**
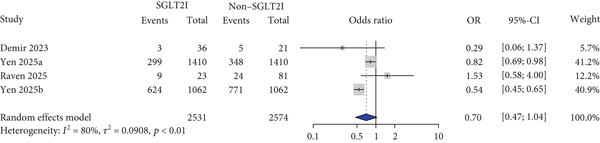
(f) All‐cause hospitalization

**Figure:**
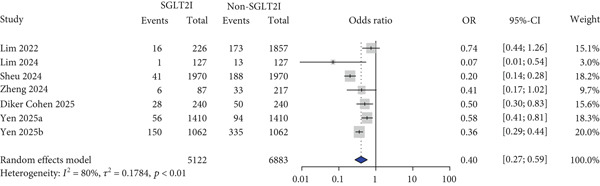
(g) All‐cause mortality

For other safety outcomes, the risks of graft rejection (OR: 0.73 and 95% CI: 0.64–0.83) (Figure 2e) and all‐cause mortality (OR: 0.40 and 95% CI: 0.27–0.59) (Figure 2g) were significantly lower in the SGLT2 inhibitor group, whereas the risk of AKI showed no significant difference between the two groups.

In the meta‐analysis based on aHRs, the evaluated outcomes showed trends consistent with those observed in the main analysis, indicating comparable efficacy and safety of SGLT2 inhibitors relative to those of control groups. Significant benefits of SGLT2 inhibitor use were observed for dialysis, MACE, all‐cause hospitalization, and all‐cause mortality (Figure S3).

#### 3.4.2. Subgroup and Sensitivity Analyses

In the subgroup analysis limited to kidney transplantation, most efficacy and safety outcomes were assessed, excluding dialysis. Overall, the trends observed in this subgroup were consistent with those in the main analysis, and the SGLT2 inhibitor group showed significant reductions in HbA1c, BMI, body weight, MACE, UTI, graft rejection, and all‐cause mortality compared with the control group (Table 2). Due to the limited number of included studies, the subgroup analysis limited to heart transplantation included only selected efficacy outcomes (excluding dialysis and cardiovascular outcomes) and safety outcomes (UTI and all‐cause hospitalization). Among these, only eGFR exhibited a significantly greater increase in the SGLT2 inhibitor group compared with the control group (MD: 5.24 and 95% CI: 0.51–9.97), whereas the other outcomes were not statistically significant (Table 3).

**Table 2:** Subgroup analysis in kidney transplantation.

**Variables**	**No. of** **studies**	**No. of participants**		**Estimates**		**95% CI**	**Heterogeneity**	
		**SGLT2I**	**Non-** **SGLT2I**				**I** ^2^	**p** **-value**
Efficacy								
HbA1c (%)	7	2,392	2,397	MD	−0.45	(−0.77, −0.13)	90%	< 0.01
Body mass index (kg/m^2^)	3	327	254	MD	−1.15	(−1.58, −0.72)	0%	0.77
Body weight (kg)	3	67	128	MD	−3.04	(−4.22, −1.85)	0%	0.59
eGFR (mL/min/1.73m^2^)	4	102	145	MD	1.71	(−2.75, 6.18)	70%	0.02
Serum creatinine (mg/dL)	2	52	66	MD	0.00	(−0.14, 0.13)	15%	0.28
Systolic blood pressure (mmHg)	3	62	126	MD	2.38	(−4.48, 9.25)	47%	0.15
Diastolic blood pressure (mmHg)	3	62	127	MD	−1.52	(−4.07, 1.03)	0%	0.96
Major adverse cardiovascular events	2	1,750	1,778	OR	0.29	(0.22, 0.38)	0%	0.91
Heart failure	2	225	197	OR	0.45	(0.08, 2.61)	0%	0.67
Myocardial infarction	2	225	197	OR	0.32	(0.02, 5.49)	69%	0.07
Safety								
Urinary tract infection	8	823	2,467	OR	0.42	(0.20, 0.85)	81%	< 0.01
Genital mycotic infection	2	166	165	OR	2.17	(0.37, 12.73)	0%	0.95
Acute kidney injury	2	284	310	OR	0.91	(0.62, 1.32)	0%	0.35
Diabetic ketoacidosis	2	284	310	OR	0.46	(0.07, 3.08)	0%	0.48
Discontinuation of SGLT2I	2	153	152	OR	13.19	(0.92, 188.40)	35%	0.21
Graft rejection	5	1,744	3,415	OR	0.72	(0.54, 0.96)	14%	0.33
All‐cause hospitalization	2	1,446	1,431	OR	0.66	(0.29, 1.51)	41%	0.19
All‐cause mortality	5	3,973	5,604	OR	0.40	(0.22, 0.73)	86%	< 0.01

Abbreviations: CI, confidence interval; eGFR, estimated glomerular filtration rate; HbA1c, hemoglobin A1c; MD, mean difference; OR, odds ratio; and SGLT2I, sodium–glucose cotransporter 2 inhibitors.

**Table 3:** Subgroup analysis in heart transplantation.

**Variables**	**No. of** **Studies**	**No. of participants**		**Estimates**		**95% CI**	**Heterogeneity**	
		**SGLT2I**	**Non-SGLT2I**				**I** ^2^	**p** **-value**
Efficacy								
HbA1c (%)	3	53	171	MD	−0.70	(−1.83, 0.42)	95%	< 0.01
Body mass index (kg/m^2^)	3	53	171	MD	−0.47	(−2.11, 1.16)	69%	0.04
Body weight (kg)	2	36	151	MD	−2.96	(−7.44, 1.52)	0%	0.95
eGFR (mL/min/1.73m^2^)	4	91	264	MD	5.24	(0.51, 9.97)	21%	0.28
Serum creatinine (mg/dL)	3	53	171	MD	−0.06	(−0.18, 0.06)	0%	0.93
Systolic blood pressure (mmHg)	2	36	151	MD	−5.73	(−18.46, 7.01)	81%	0.02
Diastolic blood pressure (mmHg)	2	36	151	MD	−1.86	(−8.07, 4.34)	72%	0.06
Safety								
Urinary tract infection	2	45	160	OR	1.26	(0.02, 82.80)	73%	0.05
All‐cause hospitalization	2	1,085	1,143	OR	0.81	(0.30, 2.20)	77%	0.04

Abbreviations: CI, confidence interval; eGFR, estimated glomerular filtration rate; HbA1c, hemoglobin A1c; MD, mean difference; OR, odds ratio; and SGLT2I, sodium–glucose cotransporter 2 inhibitors.

In the sensitivity analysis, which included only observational studies of moderate or high quality based on the risk of bias assessment, along with one RCT, the results showed a trend similar to that of the main analysis (Table 4). Significant benefits of SGLT2 inhibitor use were also observed for BMI, body weight, dialysis, MACE, HF, graft rejection, and all‐cause mortality. Leave‐one‐out sensitivity analyses revealed no influential study affecting the overall findings (Figure S4).

**Table 4:** Sensitivity analysis including one RCT and observational studies with moderate or higher quality.

**Variables**	**No. of** **Studies**	**No. of participants**		**Estimates**		**95% CI**	**Heterogeneity**	
		**SGLT2I**	**Non-** **SGLT2I**				**I** ^2^	**p** **-value**
Efficacy								
HbA1c (%)	7	2,443	2,593	MD	−0.21	(−0.42, 0.00)	73%	< 0.01
Body mass index (kg/m^2^)	4	414	471	MD	−1.13	(−1.53, −0.73)	0%	0.90
Body weight (kg)	4	154	345	MD	−3.00	(−4.11, −1.89)	0%	0.78
eGFR (mL/min/1.73m^2^)	6	208	454	MD	1.83	(−1.62, 5.28)	62%	0.02
Serum creatinine (mg/dL)	5	105	237	MD	−0.03	(−0.12, 0.05)	0%	0.79
Dialysis	2	2,472	2,472	OR	0.50	(0.31, 0.79)	74%	0.05
Systolic blood pressure (mmHg)	3	62	126	MD	2.38	(−4.48, 9.25)	47%	0.15
Diastolic blood pressure (mmHg)	3	62	127	MD	−1.52	(−4.07, 1.03)	0%	0.96
Major adverse cardiovascular events	2	1,750	1,778	OR	0.29	(0.22, 0.38)	0%	0.91
Heart failure	3	1,287	1,259	OR	0.66	(0.52, 0.83)	0%	0.83
Myocardial infarction	2	225	197	OR	0.32	(0.02, 5.49)	69%	0.07
Safety								
Urinary tract infection	8	810	2527	OR	0.48	(0.22, 1.08)	83%	< 0.01
Genital mycotic infection	2	166	165	OR	2.17	(0.37, 12.73)	0%	0.95
Acute kidney injury	2	284	310	OR	0.91	(0.62, 1.32)	0%	0.35
Diabetic ketoacidosis	2	284	310	OR	0.46	(0.07, 3.08)	0%	0.48
Discontinuation of SGLT2I	2	153	152	OR	13.19	(0.92, 188.40)	35%	0.21
Graft rejection	6	2,857	4,673	OR	0.74	(0.65, 0.84)	0%	0.58
All‐cause hospitalization	3	2,495	2,553	OR	0.74	(0.49, 1.11)	85%	< 0.01
All‐cause mortality	7	5,122	6,883	OR	0.40	(0.27, 0.59)	80%	< 0.01

Abbreviations: CI, confidence interval; eGFR, estimated glomerular filtration rate; HbA1c, hemoglobin A1c; MD, mean difference; OR, odds ratio; and SGLT2I, sodium–glucose cotransporter 2 inhibitors.

#### 3.4.3. Publication Bias Assessments

HbA1c and UTI were the outcomes of interest, which included at least 10 studies and allowed for publication bias assessments. For the HbA1c outcome, Egger′s test suggested potential publication bias (*p* = 0.04), supported by funnel plot asymmetry. Trim‐and‐fill analysis imputed five missing studies, and the effect size was no longer statistically significant after adjustment (MD: −0.10; 95% CI: −0.44 to 0.25; and *p* = 0.58), compared with the original estimate (MD: −0.59; 95% CI: −0.91 to −0.26; and *p* < 0.01). Notably, the imputed studies included effect sizes exceeding +2%, which, given the known glucose‐lowering effects of SGLT2 inhibitors, would be unlikely to occur as they imply a 2% increase in HbA1c compared with the control group. This suggests that the observed asymmetry may not represent a true publication bias. No publication bias was observed for the UTI outcome (Figure S5).

### 3.5. Efficacy and Safety Outcomes Within the SGLT2 Inhibitor Group

The use of SGLT2 inhibitors in solid organ transplant recipients led to significant reductions from baseline in HbA1c, BMI, and body weight, with MD and 95% CI as follows: −0.47% (−0.71 to −0.22) for HbA1c, −0.73 kg/m^2^ (−1.13 to −0.33) for BMI, and−2.12 kg (−3.36 to −0.87) for body weight (Table S7). However, eGFR, SCr, and blood pressure showed no significant changes from baseline. Among SGLT2 inhibitor users, the proportions of patients experiencing dialysis, MACE, HF, and MI were 5% (95% CI, 3–7%), 4% (3–5%), 5% (0–13%), and 1% (0–2%), respectively. Among the safety outcomes, the most frequently reported was all‐cause hospitalization (32%, 10–54%), followed by graft rejection (15%, 1–28%), UTI (11%, 7–14%), and AKI (16%, 3–28%) (Table S7).

## 4. Discussion

To the best of our knowledge, this is the first meta‐analysis to evaluate the efficacy and safety of SGLT2 inhibitors in patients who underwent solid organ transplant compared with a control group. The use of SGLT2 inhibitors was associated with reductions in HbA1c, body weight, and BMI. Although the number of studies was limited, SGLT2 inhibitors also appeared to reduce the risk of MACE and HF. Most safety outcomes were comparable between the groups; notably, the risks of UTI, graft rejection, and mortality were lower in the SGLT2 inhibitor group. These trends were generally maintained in subgroup analyses stratified by study quality and transplant type, although statistical significance varied in some cases. Collectively, these findings support the efficacy and safety of SGLT2 inhibitors in solid organ transplant recipients.

In this study, the magnitude of the HbA1c reduction observed in SGLT2 users (pre‐post change in single‐arm analysis, HbA1c: −0.47%) was smaller than that reported in a previous systematic review, in which combination therapy with metformin was associated with reductions of approximately 0.74%–0.8%, depending on baseline HbA1c levels [41]. This reduced effect of SGLT2 inhibitors on blood glucose levels could be explained by hyperglycemic factors, including the use of immunosuppressants such as corticosteroids and calcineurin inhibitors, in transplant recipients. However, the potential impact of immunosuppressive therapy, particularly tacrolimus and corticosteroids, on glucose metabolism could not be quantitatively evaluated because these data were inconsistently reported across the included studies. Notably, considering the comparative analysis with control groups (other antidiabetic agents), the effect of SGLT2 inhibitors on HbA1c in transplant recipients appears to be comparable to, or potentially greater than, that in controls. However, given the possibility of publication bias for this outcome, caution is warranted in interpreting the results. As obesity is a common condition among transplant recipients, the weight‐reducing effect of SGLT2 inhibitors is particularly beneficial for this population [42] and was demonstrated to be significant in our study. However, the extent of BMI reduction observed in SGLT2 users was comparable to that reported in a previous meta‐analysis of SGLT2 inhibitors, which showed a mean reduction of −0.82 kg/m^2^ (95% CI: −1.54 to −0.10) compared with that of the control group [43].

SGLT2 inhibitors are known to have cardioprotective effects in patients with diabetes, which can be attributed to their hemodynamic effects, improvements in vascular function, and positive effects on cardiac remodeling [44, 45]. Considering that PTDM is associated with increased mortality and cardiovascular events [46], evidence on the cardiovascular outcomes of SGLT2 inhibitors in transplant recipients with diabetes is clinically important. In this meta‐analysis, the risk of MACE or HF was found to be lower in the SGLT2 inhibitor group compared with the control group. Given the limited number of included studies and the fact that only Lim et al. specifically evaluated cardiovascular outcomes as the primary endpoint, the results should be interpreted with caution. However, the low heterogeneity across studies supports the validity of the pooled estimate. Furthermore, the use of propensity score matching in the included studies and the consistently observed trend toward risk reduction further strengthen the clinical relevance of this finding [28, 30, 39].

CKD, a common condition in solid organ transplant recipients, is often associated with the use of calcineurin inhibitors [47, 48]. Considering these aspects, the positive effects of SGLT2 inhibitors in patients with CKD can represent a highly attractive advantage for solid organ transplant recipients. Although no benefits in SCr levels or eGFR were observed in this study, it is important to note that the follow‐up duration in most of the included studies was less than one year, limiting the ability to evaluate the long‐term renal effects of SGLT2 inhibitors. In a subgroup analysis of heart transplant recipients, however, SGLT2 inhibitor use was associated with more favorable changes in eGFR compared with the control group. Furthermore, the risk of dialysis was significantly lower with SGLT2 inhibitor use. Although meta‐analysis of proteinuria was not feasible due to the limited number of studies and inconsistencies in measurement units, these studies demonstrated a significant reduction in proteinuria or albuminuria in the SGLT2 inhibitor group [27, 28]. Because proteinuria is a sensitive and early marker of kidney damage, these findings may have important clinical implications—even in the absence of clear improvements in eGFR. Therefore, additional well‐designed studies with longer follow‐up periods are warranted to evaluate the full spectrum of the renal effects associated with SGLT2 inhibitors in this population.

The KDIGO clinical guidelines do not recommend the use of SGLT2 inhibitors in kidney transplant recipients because of the potential risk of infection associated with SGLT2 inhibitor use. This recommendation was maintained in the revised guideline published in 2024 [10, 11]. Additionally, a systematic review conducted by Lin et al. suggested that SGLT2 inhibitor use in transplant patients might be inappropriate, considering their association with UTIs [14]. Whereas their review was limited to case series, case reports, or single‐arm studies (12 of 15), our study exclusively included comparative studies. Interestingly, the use of SGLT2 inhibitors in our study was associated with a reduced risk of UTIs compared to the control group, and this finding remained consistent in the subgroup analysis limited to kidney transplant recipients. This is notable given that UTI is a common infectious complication, particularly in kidney transplant recipients. Furthermore, UTI incidence in the SGLT2 inhibitor group (11% in kidney transplant recipients) was lower than that reported in the general diabetic population treated with SGLT2 inhibitors (approximately 34%) [49]. Moreover, the risk of genital mycotic infection did not differ significantly between the SGLT2 inhibitor and the control groups. Several clinical factors may help explain this finding. As noted in diabetes management guidelines, SGLT2 inhibitors are generally avoided or used with caution in high‐risk patients (e.g., those with recurrent UTIs or underlying urologic disorders) [50]; therefore, they tend to be prescribed to transplant recipients with relatively low baseline infection risk. In clinical practice, standardized patient education and monitoring protocols for genitourinary infections are also commonly implemented when initiating SGLT2 inhibitors, which may contribute to a lower observed infection rate. However, given the limited number of included studies [24, 28, 29], further research is warranted to clarify these safety outcomes in transplant populations, especially considering recent findings in patients with diabetes, which emphasize genital fungal infections over UTIs [51].

Lastly, this study found that the use of SGLT2 inhibitors was associated with a reduced risk of graft rejection and mortality compared with that of the control group. The reduction in rejection may be attributed to the potential immunomodulatory effects of SGLT2 inhibitors [52], which could represent a clinically meaningful benefit for transplant recipients. In addition, an in vitro study reported that empagliflozin alleviated not only tacrolimus‐induced hyperglycemia but also renal artery injury, further supporting the potential protective effects observed in clinical settings [53]. However, it should be noted that rejection was not evaluated as a primary outcome, limiting the strength of this conclusion [25, 26, 29, 40]. The observed reduction in mortality may be related to the decreased risk of graft rejection, along with the previously identified benefits of SGLT2 inhibitors on MACE, HF, and glycemic control in this study. Notably, a consistent trend toward lower mortality was observed across all included studies, and five out of the seven studies explicitly reported mortality as an outcome of interest, adding weight to the clinical relevance of this finding [26, 30–33, 39, 40]. This finding is consistent with previous evidence demonstrating a reduction in mortality risk associated with SGLT2 inhibitor use [54]. However, considering that most of the included studies were retrospective observational in nature and that current clinical guidelines do not recommend the use of SGLT2 inhibitors in solid organ transplant recipients [11], there may have been differences in the baseline clinical status between patients who received SGLT2 inhibitors and those who did not. Therefore, the possibility that such differences contributed to the observed mortality outcomes cannot be excluded, and these findings should be interpreted with caution.

This study has some limitations. First, the number of study participants in most studies was relatively small, which may have led to uncertainty in the effect estimates and limited the generalizability of the results. However, this limitation is inevitable given the characteristics of solid organ transplantation. Therefore, it may be necessary to confirm the findings of this study through larger‐scale studies, if feasible. Second, most of the studies included in this analysis were observational, which are prone to bias owing to the nature of the study design. To address this issue, we assessed the risk of bias and conducted subgroup analyses that included only studies of acceptable quality, and a consistent trend was observed. Moreover, most included studies allowed concomitant use of other hypoglycemic agents, which may have influenced glycemic outcomes. Third, most of the included studies focused on kidney or heart transplant recipients, limiting their ability to estimate the effects of SGLT2 inhibitors in liver transplant recipients. Consequently, further studies are necessary to evaluate the efficacy of SGLT2 inhibitors in liver transplant recipients. Finally, considering the patient characteristics of the included studies, these findings may not apply to transplant recipients with impaired renal function.

## 5. Conclusion

This is the first meta‐analysis to systematically evaluate the efficacy and safety of SGLT2 inhibitors with control groups in solid organ transplant recipients. SGLT2 inhibitors were associated with improvements in glycemic control and reductions in body weight, as well as favorable trends in cardiovascular outcomes. Most safety outcomes, including UTI and graft rejection, were comparable to or even more favorable in the SGLT2 inhibitor group, particularly in kidney transplant recipients. Considering the clinical significance of infections and graft rejection in transplant recipients, these findings may provide a compelling rationale for the use of SGLT2 inhibitors in this patient group. However, given the limited number of studies available for certain outcomes such as cardiovascular events, further large‐scale comparative studies are required to confirm these findings and to evaluate the long‐term effects of SGLT2 inhibitors in diverse transplant populations.

## Disclosure

All authors participated in the final approval of the manuscript.

## Conflicts of Interest

The authors declare no conflicts of interest.

## Author Contributions

All authors participated in the conception and design of the study, the data acquisition and analysis, the interpretation of data, and the writing of the manuscript.

## Funding

This work was supported by the 2024 Yeungnam University Research Grant (224A061021).

## Supporting information


**Supporting Information** Additional supporting information can be found online in the Supporting Information section. Table S1: Checklist for preferred reporting items for systematic reviews and meta‐analyses. Table S2: Search strategy. Table S3: The detailed characteristics of the included patients. Table S4: The detailed study design and outcomes of the included studies. Table S5: Detailed information regarding the risk of bias assessment according to the Risk of Bias 2 criteria. Table S6: Summary of the meta‐analysis results for other efficacy outcomes of SGLT2 inhibitors with the control groups. Table S7: Summary of the meta‐analysis results for efficacy and safety outcomes within the SGLT2 group. Figure S1: Flowchart of the study selection process. Figure S2: Quality assessment of the risk of bias according to the ROBINS‐I criteria. Figure S3: Forest plots of hazard ratios. Figure S4: Sensitivity analysis with the leave‐one‐out method. Figure S5: Funnel plots.

## Data Availability

All data generated or analyzed during this study are included in this published article and its supporting information.
