# Supplementary material for: Sodium–Glucose Cotransporter 2 Inhibitors in Diabetic Solid Organ Transplant Recipients: A Systematic Review and Meta‐Analysis of Comparative Studies
Source: J Diabetes Res. 2026 Jan 19;2026:8540354. doi: 10.1155/jdr/8540354 (PMC12814960; doi:10.1155/jdr/8540354)
Supplement: Supplementary file 1 — Supporting Information Additional supporting information can be found online in the Supporting Information section. Table S1: Checklist for preferred reporting items for systematic reviews and meta‐analyses. Table S2: Search strategy. Table S3: The detailed characteristics of the included patients. Table S4: The detailed study design and outcomes of the included studies. Table S5: Detailed information regarding the risk of bias assessment according to the Risk of Bias 2 criteria. Table S6: Summary of the meta‐analysis results for other efficacy outcomes of SGLT2 inhibitors with the control groups. Table S7: Summary of the meta‐analysis results for efficacy and safety outcomes within the SGLT2 group. Figure S1: Flowchart of the study selection process. Figure S2: Quality assessment of the risk of bias according to the ROBINS‐I criteria. Figure S3: Forest plots of hazard ratios. Figure S4: Sensitivity analysis with the leave‐one‐out method. Figure S5: Funnel plots. [file JDR-2026-8540354-s001.docx]

**The efficacy and safety of sodium-glucose cotransporter-2 inhibitors in solid organ transplant recipients with diabetes mellitus: a systematic review and meta-analysis of comparative studies**

**Supplementary Materials**

Table S1. Checklist for Preferred Reporting Items for Systematic Reviews and Meta-Analyses

Table S2. Search strategy

Table S3. The detailed characteristics of the included patients

Table S4. The detailed study design and outcomes of the included studies

Table S5. Detailed information regarding the risk of bias assessment according to the Risk of Bias 2 criteria

Table S6. Summary of the meta-analysis results for other efficacy outcomes of SGLT2 inhibitors with the control groups

Table S7. Summary of the meta-analysis results for efficacy and safety outcomes within the SGLT2 group

Fig. S1 Flowchart of the study selection process

Fig. S2 Quality assessment of the risk of bias according to the ROBINS-I criteria

Fig. S3 Forest plots of hazard ratios

Fig. S4 Sensitivity analysis with the leave-one-out method

Fig. S5 Funnel plots

Table S1. Checklist for Preferred Reporting Items for Systematic Reviews and Meta-Analyses

| **Section/topic** | **#** | **Checklist item** | **Reported on page #** |
| --- | --- | --- | --- |
| **TITLE** |  |  |  |
| Title | 1 | Identify the report as a systematic review, meta-analysis, or both. | 1 |
| **ABSTRACT** |  | |  |
| Structured summary | 2 | Provide a structured summary including, as applicable: background; objectives; data sources; study eligibility criteria, participants, and interventions; study appraisal and synthesis methods; results; limitations; conclusions and implications of key findings; systematic review registration number. | 3-4 |
| **INTRODUCTION** |  |  |  |
| Rationale | 3 | Describe the rationale for the review in the context of what is already known. | 5-7 |
| Objectives | 4 | Provide an explicit statement of questions being addressed with reference to participants, interventions, comparisons, outcomes, and study design (PICOS). | 7 |
| **METHODS** | | | |
| Protocol and registration | 5 | Indicate if a review protocol exists, if and where it can be accessed (e.g., Web address), and, if available, provide registration information including registration number. | 7 |
| Eligibility criteria | 6 | Specify study characteristics (e.g., PICOS, length of follow-up) and report characteristics (e.g., years considered, language, publication status) used as criteria for eligibility, giving rationale. | 7 |
| Information sources | 7 | Describe all information sources (e.g., databases with dates of coverage, contact with study authors to identify additional studies) in the search and date last searched. | 7 |
| Search | 8 | Present full electronic search strategy for at least one database, including any limits used, such that it could be repeated. | 7, and  Table S2 |
| Study selection | 9 | State the process for selecting studies (i.e., screening, eligibility, included in systematic review, and, if applicable, included in the meta-analysis). | 7-8 |
| Data collection process | 10 | Describe method of data extraction from reports (e.g., piloted forms, independently, in duplicate) and any processes for obtaining and confirming data from investigators. | 8-9 |
| Data items | 11 | List and define all variables for which data were sought (e.g., PICOS, funding sources) and any assumptions and simplifications made. | 8-9 |
| Risk of bias in individual studies | 12 | Describe methods used for assessing risk of bias of individual studies (including specification of whether this was done at the study or outcome level), and how this information is to be used in any data synthesis. | 10 |
| Summary measures | 13 | State the principal summary measures (e.g., risk ratio, difference in means). | 9 |
| Synthesis of results | 14 | Describe the methods of handling data and combining results of studies, if done, including measures of consistency (e.g., I^2^) for each meta-analysis. | 9 |
| Risk of bias across studies | 15 | Specify any assessment of risk of bias that may affect the cumulative evidence (e.g., publication bias, selective reporting within studies). | 11 |
| Additional analyses | 16 | Describe methods of additional analyses (e.g., sensitivity or subgroup analyses, meta-regression), if done, indicating which were pre-specified. | 9-10 |
| **RESULTS** | | | |
| Study selection | 17 | Give numbers of studies screened, assessed for eligibility, and included in the review, with reasons for exclusions at each stage, ideally with a flow diagram. | 10-11 and  Fig. S1 |
| Study characteristics | 18 | For each study, present characteristics for which data were extracted (e.g., study size, PICOS, follow-up period) and provide the citations. | 11,  Table 1,  and S3 |
| Risk of bias within studies | 19 | Present data on risk of bias of each study and, if available, any outcome level assessment (see item 12). | 12, Table S5, and Fig. S2 |
| Results of individual studies | 20 | For all outcomes considered (benefits or harms), present, for each study: (a) simple summary data for each intervention group (b) effect estimates and confidence intervals, ideally with a forest plot. | 11-15, and Table S4 |
| Synthesis of results | 21 | Present results of each meta-analysis done, including confidence intervals and measures of consistency. | 11-15,  Table S6, Fig. 1, 2, and S3 |
| Risk of bias across studies | 22 | Present results of any assessment of risk of bias across studies (see Item 15). | 14-15, Figure S5 |
| Additional analysis | 23 | Give results of additional analyses, if done (e.g., sensitivity or subgroup analyses, meta-regression [see Item 16]). | 13-15,  Table 2-4, S7 and Fig. S4 |
| **DISCUSSION** |  |  |  |
| Summary of evidence | 24 | Summarize the main findings including the strength of evidence for each main outcome; consider their relevance to key groups (e.g., healthcare providers, users, and policy makers). | 15-20 |
| Limitations | 25 | Discuss limitations at study and outcome level (e.g., risk of bias), and at review-level (e.g., incomplete retrieval of identified research, reporting bias). | 20-21 |
| Conclusions | 26 | Provide a general interpretation of the results in the context of other evidence, and implications for future research. | 21-22 |
| **FUNDING** |  |  |  |
| Funding | 27 | Describe sources of funding for the systematic review and other support (e.g., supply of data); role of funders for the systematic review. | 22 |

Table S2. Search strategy

| **MEDLINE** | |
| --- | --- |
| #1 | "kidney"[Title/Abstract] OR "renal"[Title/Abstract]) AND ("transplant*"[Title/Abstract] OR "graft*"[Title/Abstract] OR "allograft*"[Title/Abstract] OR "homograft*"[Title/Abstract] OR "recipient*"[Title/Abstract] |
| #2 | "liver"[Title/Abstract] OR "hepatic"[Title/Abstract]) AND ("transplant*"[Title/Abstract] OR "graft*"[Title/Abstract] OR "allograft*"[Title/Abstract] OR "homograft*"[Title/Abstract] OR "recipient*"[Title/Abstract] |
| #3 | "heart"[Title/Abstract] OR "cardiac"[Title/Abstract]) AND ("transplant*"[Title/Abstract] OR "graft*"[Title/Abstract] OR "allograft*"[Title/Abstract] OR "homograft*"[Title/Abstract] OR "recipient*"[Title/Abstract] |
| #4 | "lung"[Title/Abstract] OR "pulmonary"[Title/Abstract]) AND ("transplant*"[Title/Abstract] OR "graft*"[Title/Abstract] OR "allograft*"[Title/Abstract] OR "homograft*"[Title/Abstract] OR "recipient*"[Title/Abstract] |
| #5 | "sodium glucose transporter 2 inhibitor*"[Title/Abstract] OR "sglt 2 inhibitor*"[Title/Abstract] OR "sglt2 inhibitor*"[Title/Abstract] OR "sodium glucose cotransporter 2 inhibitor*"[Title/Abstract] OR "sodium glucose co transporter 2 inhibitor*"[Title/Abstract] OR "sodium dependent glucose cotransporter 2 inhibitor*"[Title/Abstract] OR "gliflozin*"[Title/Abstract] OR "Canagliflozin"[Title/Abstract] OR "Invokana"[Title/Abstract] OR "Canaglu"[Title/Abstract] OR "Motivyst"[Title/Abstract] OR "Sulisent"[Title/Abstract] OR "Prominad"[Title/Abstract] OR "Dapagliflozin"[Title/Abstract] OR "Farxiga"[Title/Abstract] OR "Forxiga"[Title/Abstract] OR "Forziga"[Title/Abstract] OR "Edistride"[Title/Abstract] OR "Oxra"[Title/Abstract] OR "Empagliflozin"[Title/Abstract] OR "Jardiance"[Title/Abstract] OR "Gibulio"[Title/Abstract] OR "Ertugliflozin"[Title/Abstract] OR "Steglatro"[Title/Abstract] OR "Ipragliflozin"[Title/Abstract] OR "Suglat"[Title/Abstract] OR  "sotagliflozin"[Title/Abstract] OR "Inpefa"[Title/Abstract] OR "zynquista"[Title/Abstract] OR "luseogliflozin"[Title/Abstract] OR "Lusefi"[Title/Abstract] OR "Bexagliflozin"[Title/Abstract] OR "Brenzavvy"[Title/Abstract] OR "Enavogliflozin"[Title/Abstract] OR "Henagliflozin"[Title/Abstract] OR "Janagliflozin"[Title/Abstract] OR "JP-2266"[Title/Abstract] OR "Mizagliflozin"[Title/Abstract] OR "Rongliflozin"[Title/Abstract] OR "Tofogliflozin"[Title/Abstract] OR "licogliflozin"[Title/Abstract] OR "remogliflozin etabonate"[Title/Abstract] OR "sergliflozin etabonate"[Title/Abstract] |
| #6 | (#1 OR #2 OR #3 OR #4) AND #5 |
| #7 | "animals"[MeSH Terms] NOT "humans"[MeSH Terms] |
| #8 | #6 NOT #7 |
| **Embase** | |
| #1 | 'kidney transplant*':ab,ti or 'kidney allotransplant*':ab,ti or 'kidney homotransplant*':ab,ti or 'kidney retransplant*':ab,ti or 'kidney graft*':ab,ti or 'kidney allograft*':ab,ti or 'kidney homograft*':ab,ti or 'kidney recipient*':ab,ti or 'renal transplant*':ab,ti or 'renal allotransplant*':ab,ti or 'renal homotransplant*':ab,ti or 'renal retransplant*':ab,ti or 'renal graft*':ab,ti or 'renal allograft*':ab,ti or 'renal homograft*':ab,ti or 'renal recipient*':ab,ti |
| #2 | 'liver transplant*':ab,ti or 'liver allotransplant*':ab,ti or 'liver homotransplant*':ab,ti or 'liver retransplant*':ab,ti or 'liver graft*':ab,ti or 'liver allograft*':ab,ti or 'liver homograft*':ab,ti or 'liver recipient*':ab,ti or 'hepatic transplant*':ab,ti or 'hepatic allotransplant*':ab,ti or 'hepatic homotransplant*':ab,ti or 'hepatic retransplant*':ab,ti or 'hepatic graft*':ab,ti or 'hepatic allograft*':ab,ti or 'hepatic homograft*':ab,ti or 'hepatic recipient*':ab,ti |
| #3 | 'heart transplant*':ab,ti or 'heart allotransplant*':ab,ti or 'heart homotransplant*':ab,ti or 'heart retransplant*':ab,ti or 'heart graft*':ab,ti or 'heart allograft*':ab,ti or 'heart homograft*':ab,ti or 'heart recipient*':ab,ti or 'cardiac transplant*':ab,ti or 'cardiac allotransplant*':ab,ti or 'cardiac homotransplant*':ab,ti or 'cardiac retransplant*':ab,ti or 'cardiac graft*':ab,ti or 'cardiac allograft*':ab,ti or 'cardiac homograft*':ab,ti or 'cardiac recipient*':ab,ti |
| #4 | 'lung transplant*':ab,ti or 'lung allotransplant*':ab,ti or 'lung homotransplant*':ab,ti or 'lung retransplant*':ab,ti or 'lung graft*':ab,ti or 'lung allograft*':ab,ti or 'lung homograft*':ab,ti or 'lung recipient*':ab,ti or 'pulmonary transplant*':ab,ti or 'pulmonary allotransplant*':ab,ti or 'pulmonary homotransplant*':ab,ti or 'pulmonary retransplant*':ab,ti or 'pulmonary graft*':ab,ti or 'pulmonary allograft*':ab,ti or 'pulmonary homograft*':ab,ti or 'pulmonary recipient*':ab,ti |
| #5 | 'sodium glucose transporter 2 inhibitor*':ab,ti OR 'sglt 2 inhibitor*':ab,ti OR 'sglt2 inhibitor*':ab,ti OR 'sodium glucose cotransporter 2 inhibitor*':ab,ti OR 'sodium glucose co transporter 2 inhibitor*':ab,ti OR 'sodium dependent glucose cotransporter 2 inhibitor*':ab,ti OR 'gliflozin*':ab,ti OR 'Canagliflozin':ab,ti OR 'Invokana':ab,ti OR 'Canaglu':ab,ti OR 'Motivyst':ab,ti OR 'Sulisent':ab,ti OR 'Prominad':ab,ti OR 'Dapagliflozin':ab,ti OR 'Farxiga':ab,ti OR 'Forxiga':ab,ti OR 'Forziga':ab,ti OR 'Edistride':ab,ti OR 'Oxra':ab,ti OR 'Empagliflozin':ab,ti OR 'Jardiance':ab,ti OR 'Gibulio':ab,ti OR 'Ertugliflozin':ab,ti OR 'Steglatro':ab,ti OR 'Ipragliflozin':ab,ti OR 'Suglat':ab,ti OR  'sotagliflozin':ab,ti OR 'Inpefa':ab,ti OR 'zynquista':ab,ti OR 'luseogliflozin':ab,ti OR 'Lusefi':ab,ti OR 'Bexagliflozin':ab,ti OR 'Brenzavvy':ab,ti OR 'Enavogliflozin':ab,ti OR 'Henagliflozin':ab,ti OR 'Janagliflozin':ab,ti OR 'JP-2266':ab,ti OR 'Mizagliflozin':ab,ti OR 'Rongliflozin':ab,ti OR 'Tofogliflozin':ab,ti OR 'licogliflozin':ab,ti OR 'remogliflozin etabonate':ab,ti OR 'sergliflozin etabonate':ab,ti |
| #6 | (#1 OR #2 OR #3 OR #4) AND #5 |
| #7 | ('animal':de OR 'animal experiment':de OR 'nonhuman':de) NOT ('human':de OR 'human experiment':de) |
| #8 | #6 NOT #7 |
| **CENTRAL** | |
| #1 | ((kidney transplant*) or (kidney allotransplant*) or (kidney homotransplant*) or (kidney retransplant*) or (kidney graft*) or (kidney allograft*) or (kidney homograft*) or (kidney recipient*) or (renal transplant*) or (renal allotransplant*) or (renal homotransplant*) or (renal retransplant*) or (renal graft*) or (renal allograft*) or (renal homograft*) or (renal recipient*)):ti,ab,kw |
| #2 | ((liver transplant*) or (liver allotransplant*) or (liver homotransplant*) or (liver retransplant*) or (liver graft*) or (liver allograft*) or (liver homograft*) or (liver recipient*) or (hepatic transplant*) or (hepatic allotransplant*) or (hepatic homotransplant*) or (hepatic retransplant*) or (hepatic graft*) or (hepatic allograft*) or (hepatic homograft*) or (hepatic recipient*)):ti,ab,kw |
| #3 | ((heart transplant*) or (heart allotransplant*) or (heart homotransplant*) or (heart retransplant*) or (heart graft*) or (heart allograft*) or (heart homograft*) or (heart recipient*) or (cardiac transplant*) or (cardiac allotransplant*) or (cardiac homotransplant*) or (cardiac retransplant*) or (cardiac graft*) or (cardiac allograft*) or (cardiac homograft*) or (cardiac recipient*)):ti,ab,kw |
| #4 | ((lung transplant*) or (lung allotransplant*) or (lung homotransplant*) or (lung retransplant*) or (lung graft*) or (lung allograft*) or (lung homograft*) or (lung recipient*) or (pulmonary transplant*) or (pulmonary allotransplant*) or (pulmonary homotransplant*) or (pulmonary retransplant*) or (pulmonary graft*) or (pulmonary allograft*) or (pulmonary homograft*) or (pulmonary recipient*)):ti,ab,kw |
| #5 | ((sodium glucose transporter 2 inhibitor*) or (sglt 2 inhibitor*) or (sglt2 inhibitor*) or (sodium glucose cotransporter 2 inhibitor*) or (sodium glucose co transporter 2 inhibitor*) or (sodium dependent glucose cotransporter 2 inhibitor*) or (gliflozin*) or (Canagliflozin) or (Invokana) or (Canaglu) or (Motivyst) or (Sulisent) or (Prominad) or (Dapagliflozin) or (Farxiga) or (Forxiga) or (Forziga) or (Edistride) or (Oxra) or (Empagliflozin) or (Jardiance) or (Gibulio) or (Ertugliflozin) or (Steglatro) or (Ipragliflozin) or (Suglat) or (sotagliflozin) or (Inpefa) or (zynquista) or (luseogliflozin) or (Lusefi) or (Bexagliflozin) or (Brenzavvy) or (Enavogliflozin) or (Henagliflozin) or (Janagliflozin) or (JP-2266) or (Mizagliflozin) or (Rongliflozin) or (Tofogliflozin) or (licogliflozin) or (remogliflozin etabonate) or (sergliflozin etabonate)):ti,ab,kw |
| #6 | (#1 OR #2 OR #3 OR #4) AND #5 |

Table S3. The detailed characteristics of the included patients.

| **Author, year  (country)** | **PTDM,**  **%** | **Duration**  **of DM,**  **year,**  **mean** | **Duration**  **post**  **transplant,**  **year, mean** | **Living donor, %** | **ABO  incompa-tibility, %** | **Immunosuppressants  (maintenance therapy)** | **Comorbidities** |
| --- | --- | --- | --- | --- | --- | --- | --- |
| ***Kidney Transplantation*** | |  |  |  |  |  |  |
| Halden, 2019  (Norway) [24] | 100 | N/A | 3^a^ | 36.3 | N/A | Tacrolimus 79.6%  (median trough level *I:* 5.4 ng/ml, *C:* 6.2 ng/ml) Cyclosporine 13.6% Mycophenolate 91.0% Everolimus 4.6% Prednisone 97.8% | N/A |
| Hidasome, 2021 (Japan) [50] | 0 | N/A | N/A | N/A | 40.0 | Triple-combination therapy: Tacrolimus (97.8%) or cyclosporine (2.2%)  + Mycophenolate mofetil (68.5%) or everolimus (31.5%)  + Methylprednisolone | CVD 39.30% |
| Lim, 2022  (South Korea) [49] | 22.8 | N/A | N/A | *I:* 78.3* *C:* 70.3* | 14.7 | Tacrolimus 81.9%  (mean trough level *I:* 6.4 ng/ml, *C:* 5.9 ng/ml)* Cyclosporine 19.5% (*I:* 11.9%, *C:* 20.4%)* Steroids 97.9% | Hypertension 76.8% Dyslipidemia 7.3% Ischemic heart disease 12.1%  Cerebrovascular disease 8.0% |
| Demir, 2023  (Turkey) [43] | *I:* 61.1* *C:* 33.3* | 14.8 | *I:* 4.9^a^* *C:* 1.5^a^* | 94.7 | N/A | Triple-combination therapy: Prednisolone + Mycophenolic acid + Calcineurin inhibitor (most commonly tacrolimus) | CVD 47.4% |
| Mahmoud, 2023 (Kuwait) [27] | 44.6 | N/A | 7.4 | 75.6 | N/A | Cyclosporin 18.5% Tacrolimus 75.6% None 6.0% Other: N/A | N/A |
| Yeggalam, 2023 (USA) [28] | 46.5 | N/A | 7.1 | 14.9 | N/A | N/A | N/A |
| Lim, 2024  (South Korea) [40] | 16.9 | N/A | N/A | 78.7 | 20.1 | Tacrolimus 87.0% Cyclosporine 15.0% Other: N/A | Hypertension 81.5% Dyslipidemia 11.0% Ischemic heart disease 13.0% |
| Sheu, 2024 (Taiwan) [31] | N/A | N/A | N/A | N/A | N/A | N/A | Hypertension 86.5% Dyslipidemia 70.4%  Heart failure 21.4% |
| Diker Cohen, 2025 (Israel) [51] | *I:*43.0  *C:*46.0 | N/A | N/A | *I:*76.0%  *C:*60.0% | N/A | Tacrolimus 87.8%  Cyclosporine 5.9% Immunomodulators 82.3%  Everolimus 8.4% Sirolimus 2.1% Prednisone 97.7% | Hypertension 93.3% Dyslipidemia 87.3%  Ischemic heart disease 43.3%  Cerebrovascular event 18.3%  Congestive heart failure 14.6%^*^ |
| Yen, 2025a (Taiwan) [30] | N/A | N/A | N/A | N/A | N/A | Tacrolimus 56.4%  Cyclosporine 5.6% Mycopheonolate 3.1%  Everolimus 1.3%  Corticosteroids 60.8% | Hypertension 79.8% Dyslipidemia 58.1%  Ischemic heart disease 26.7%  Cerebrovascular disease 8.8% |
| ***Heart Transplantation*** | |  |  |  |  |  |  |
| Muir, 2017  (Austrailia) [25] | N/A | 8.4 | 9.4 | 0 | N/A | Prednisone 55.6% Other: N/A | Hypertension 91.1% |
| Cehic, 2019  (Austrailia) [26] | 55.4 | *I*: 8.0^a^ *C*: 6.0^a^ | *I*: 5.0^a^ *C*: 8.0^a^ | 0 | N/A | Tacrolimus 39.6% Cyclosporine 30.7% Everolimus 44.6% Prednisolone 47.5% | Hypertension 89.1% Hypercholesterolemia 94.1% |
| Marfella, 2022 (Italy) [53] | 0 | N/A | N/A | 0 | N/A | Tacrolimus or cyclosporine, mycophenolate mofetil, everolimus, and prednisone | Hypertension 13.5% Dyslipidemia 43.2% |
| Lu, 2025 (Taiwan) [54] | N/A | N/A | *I*: 9.1 *C*: 7.2 | 0 | N/A | Tacrolimus 63.5%  Cyclosporine 36.5% Mycophenolate 92.9%  Azathioprine 3.5%  Everolimus 12.9% Prednisone 80.0% | Hypertension 57.6% |
| Raven, 2025 (Australia) [32] | *I*: 30.0 *C*: 72.0 | N/A | N/A | 0 | N/A | Tacrolimus (trough level 6~10 mg/L),  mycophenolate (1,000 mg bid), and prednisolone  Everolimus 65.4% | N/A |
| Yen, 2025b (Taiwan) [29] | N/A | N/A | N/A | 0 | N/A | Tacrolimus 44.3%  Cyclosporine 4.7% Everolimus 1.3% Prednisone 33.3% | Hypertension 84.2% Dyslipidemia 72.5%  Ischemic heart disease 55.6%  Cerebrovascular disease 22.7%  Heart failure 66.2% |
| ***Liver Transplantation*** | |  |  |  |  |  |  |
| Zheng, 2024  (Canada) [33] | *I*: 54.0* *C*: 69.1* | N/A | *I*: 9.8^a^* *C*: 6.4^a^* | *I*: 40.2* *C*: 19.8* | N/A | Tacrolimus 80.2% (trough level 2~17.6 mg/L) Cyclosporine 35.9% Sirolimus 16.8% Other: N/A | N/A |

a median

* indicates a significant difference between the intervention group and control group.

PTDM: posttransplant diabetes mellitus, DM: diabetes mellitus, CVD: cardiovascular disease, *C*: control group, *I*: intervention group

Table S4. The detailed study design and outcomes of the included studies.

| **Author, year  (country)** | **Inclusion** | **Exclusion** | **Adjustment for confounder; variables** | **Endpoints** |
| --- | --- | --- | --- | --- |
| ***Kidney Transplantation*** | |  |  | |
| Halden, 2019  (Norway) [24] | - Aged ≥18 years - PTDM with persistent hyperglycemia for at least 1 year after KT - Transplanted ≥1 year prior to inclusion - Stable renal function  (<20% deviation in serum creatinine within the last 2 months) - Stable immunosuppressive therapy for at least 3 months before inclusion - Provided written informed consent | - Pre-transplant DM - eGFR <30 mL/min/1.73 m^2^ - Pregnant or nursing mothers | - Randomized, placebo controlled, and double blinded   ; Groups were well matched according to age, sex, body weight, and comorbidities. | - Primary endpoint   : 24-weeks change in weighted mean glucose estimated with continuous glucose monitoring system   - Secondary endpoint   : 24-weeks change in  HbA1c, FPG, 2h plasma glucose after OGTT, body weight, WHR, VAT, BP, and eGFR   - Other outcomes: adverse events including UTI, genital infection, and graft rejection |
| Hidasome, 2021 (Japan) [50] | - Pre-transplant T2DM as the primary cause of ESRD - Newly administered OHAs during post-transplant follow-up | - Patients followed up at other institutions - Observation period <1 year - Patients with missing data on variables | - Inverse probability of treatment weighting (IPTW)   ; recipient age, sex, BMI, history of cardiovascular disease, ABO incompatibility, insulin therapy, eGFR, and HbA1c at the initiation of additional OHAs. | - Primary endpoint: 1-y changes in HbA1c, body weight, and eGFR - Secondary endpoint: 1-y changes in BP, total cholesterol, LDL, HDL, triglycerides - Other outcomes: adverse events, and biopsy-proven acute rejection |
| Lim, 2022  (South Korea) [49] | - Pre-transplant DM or PTDM | - History of pancreas transplantation - SGLT2i use <90 days | - Propensity score matching   ; age, sex, body mass index, donor type, ABO incompatibility, acute rejection, comorbid diabetes, hypertension, dyslipidemia, diabetic ESRD, ACE inhibitor or ARB usage, eGFR at 3 months after transplant, mean HbA1c for 1 year after transplantation, and metformin usage. | - Primary endpoint: a composite outcome of all-cause mortality, death-censored graft failure (DCGF), and serum creatinine doubling. - Secondary endpoint: all-cause mortality, DCGF, serum creatinine doubling, eGFR, acute eGFR dip, adverse events including UTI, urogenital fungal infection, and euglycemic ketoacidosis. - Other clinical outcomes: serum creatinine and HbA1c at 3 months and 1 year after KT. |
| Demir, 2023  (Turkey) [43] | - Pre-transplant T2DM or PTDM | - SGLT2i use <6 months | N/A | - Development of UTI, genital infection, acute rejection, and euglycemic diabetic ketoacidosis - Change of body weight, serum creatinine, eGFR, proteinuria, HbA1c |
| Mahmoud, 2023 (Kuwait) [27] | - Pre-transplant T2DM or PTDM - Stable renal function for at least 3 months - eGFR > 25 ml/min/1.73m^2^ | - Recurrent UTI - Persistent gastrointestinal symptoms. | - Case-control matching   ; age, sex, BMI, type of donor, post-transplant duration, immunosuppression, and baseline eGFR. | - Efficacy: changes in HbA1c and BMI over the year, the changes of eGFR and U-ACR, the changes in systolic and diastolic BP. - Safety: incidence of UTI, genital infection, MI, HF, CVA, and drug discontinuation due to side effects. |
| Yeggalam, 2023 (USA) [28] | - Pre-transplant DM or PTDM | - SGLT2i use <1 year | - Propensity score matching   ; log-transformed age, type of transplant, duration of transplantation and baseline eGFR. | - Primary endpoint: changes in eGFR or serum creatinine at 1 year. - Secondary endpoint: hospitalizations with diagnoses of acute kidney injury, diabetic ketoacidosis, cellulitis and amputation, acute transplant rejection confirmed by kidney biopsies, UTI and genital mycotic infection, body weight, BP, HbA1c, cholesterol, LDL, HDL, triglycerides and urinary protein/creatinine excretion. |
| Lim, 2024  (South Korea) [40] | - Pre-transplant DM or PTDM | - History of pancreas transplantation - SGLT2i use <90 days - Heart failure, acute coronary syndrome, or stroke and had received PCI or coronary artery bypass surgery within 3 months before KT | - Propensity score matching   ; age, sex, body mass index, donor type, ABO-incompatibility, use of angiotensin-converting enzyme inhibitor or angiotensin II receptor blocker, and 1-year mean HbA1c level. | - Primary endpoint: MACE consisting of MI, death from cardiovascular causes, hospitalization for heart failure, or stroke - Secondary endpoint: Each component of MACE - Other clinical outcomes: serum creatinine and HbA1c at 3 months and 1 year after KT, incidence of UTI and euglycemic ketoacidosis |
| Sheu, 2024 (Taiwan) [31] | - Aged >18 years - KT recipients with type 2 DM - SGLT2i group: SGLT2i initiated within 3 months after KT - Control group: non-SGLT2i users within 3 months after KT | - Died or required dialysis between 1 to 3 months after transplantation | - Propensity score matching (1:1)   ; 45 characteristics(demographics, medical history, concomitant medications, laboratory test results)   - Multivariable Cox proportional hazards model - Adjusted variables: age, gender, and race | - Outcome evaluation was done from the 90th day after transplantation and up to 5 years - Primary outcomes: all-cause mortality - Secondary outcomes: MACE, MAKE - MACE: cerebral infarction, hemorrhagic stroke, AMI, cardiac arrest, and death - MAKE: initiation of re-dialysis, incident dialysis, or death - Other: AKI, genitourinary infections (UTI + candidiasis), UTI, urogenital candidiasis |
| Diker Cohen, 2025 (Israel) [51] | - Aged ≥18 years - Pre-transplant DM or PTDM | - Type 1 DM - eGFR < 30 ml/min/1.73m^2^ - on dialysis | - SGLT2i and control groups were matched 1:1 by gender and age at first kidney transplantation - In the multivariate Cox regression analysis, variables that were statistically significant but not strongly associated were included as independent variables. | - Metabolic effects: HbA1c, BMI - Safety profile - Renal-related: hospital admission for UTIs, composite of dialysis, re-transplantation, AKI, or acute rejection - Diabetic ulcers, fractures, lower limb amputations, DKA, all-cause mortality |
| Yen, 2025a (Taiwan) [30] | - Pre-transplant DM or PTDM - SGLT2i group: at least 1 SGLT2i prescription within 3 months after KT - DPP-4i group: at least 1 DPP-4i prescription within 3 months after KT | - Aged < 20 years - DM without antidiabetic medications - Type 1 DM - Medication changes between SGLT2i and DPP-4i groups during follow-up | - Propensity score matching (1:1)   ; age, sex, race, socioeconomic status, lifestyle factors, comorbidities, medications, and laboratory results. | - Sepsis or infection - Transplant rejection - Need for dialysis - All-cause hospitalization - All-cause mortality |
| ***Heart Transplantation*** | |  |  | |
| Muir, 2017  (Austrailia) [25] | - DM - Patients with follow-up after a minimum period of 3 months of empagliflozin therapy or other DM treatment | N/A | N/A | - Changes in body weight, BMI, BP, HbA1c, diuretic (furosemide) dose, and renal function. - Adverse events potentially attributable to empagliflozin (genitourinary infections, etc.) |
| Cehic, 2019  (Austrailia) [26] | - T2DM or PTDM - Patients with follow-up after a minimum period of 12 months of empagliflozin therapy or other DM treatment | N/A | N/A | - Primary outcome: prevalence of adverse effects attributable to empagliflozin, with genitourinary infection. - Secondary outcome: body weight, BMI, BP, HbA1c, diuretic usage (furosemide dose), and renal function. |
| Marfella, 2022 (Italy) [53] | - T2DM for at least 6 months before HT, with optimal glycemic control (HbA1c <7%) and without diabetic complications.   - Patients who had already used SGLT2 inhibitors for at least 6 months before HT and continued throughout the follow-up  (SGLT2i diabetic group)   - Patients who never used SGLT2i (no-SGLT2i diabetic group) - Absence of pre-transplantation T2DM (non-diabetic group) | - Patients with endomyocardial biopsy specimens consistent with ISHLT grade 2R considered positive for rejection - Positive donor-specific antibodies - Increased T4/T8 ratio - Positive IgM and/or IgG cytomegalovirus antibodies - PTDM | N/A | - Clinical (BMI, HbA1c, serum creatinine) and echocardiographic evaluations - Heart biopsies and tissue analysis:  JunD expression, IRS1 and IRS2, PPAR-γ, ceramide levels |
| Lu, 2025 (Taiwan) [54] | - HT patients with DM - eGFR > 30 ml/min/1.73m2 - SGLT2i group: Continuous users of SGLT2i (>= 1year) | - N/A | - Propensity score matching: eGFR, hypertension, insulin use - Multivariable Cox proportional hazards model   Adjusted variables: age, gender, time since TPL, eGFR at baseline, HbA1c at baseline, calcineurin inhibitors, hypertension, SBP, DBP | - Renal-specific composite outcome: a decline of at least 50% in eGFR, the onset of ESRD, death from renal causes |
| Raven, 2025 (Australia) [32] | - HT patients with DM or PTDM - SGLT2i group: SGLT2i initiated within 12 months after HT and continued for at least 6 months | - Co-transplantation with a kidney - Dialysis from the time transplant | - Mortality: multivariable Cox proportional hazards model   Adjusted variables: diabetes type, baseline creatinine | - Primary outcomes: difference in eGFR change from baseline, assessed at 3 years post-HT - Mortality |
| Yen, 2025b (Taiwan) [29] | - HT patients with DM - SGLT2i group: at least 1 SGLT2i prescription | - Aged < 20 years - DM without antidiabetic medications - Type 1 DM - Dialysis - Received KT - Death before index dates | - Propensity score matching (1:1)   ; age, sex, race, BMI, lifestyle or environmental factors, comorbidities, medications, and laboratory results. | - Heart transplant failure and rejection - Post-transplant sepsis or infection - Need for dialysis - Renal replacement therapy - Kidney transplantation - Ischemic heart disease, heart failure exacerbation - All-cause hospitalization - All-cause mortality |
| ***Liver Transplantation*** | |  |  | |
| Zheng, 2024  (Canada) [33] | - Aged >18 years - T2DM or PTDM - Started one or both of GLP-1RAs and SGLT2 inhibitors (intervention groups) or DPP-4 inhibitors (comparator group) at least 3 months after LT | - Type 1 diabetes | - Multivariable linear mixed models - Included risk factors: time since drug initiation (at 3, 6 or 12 months), medication group, time and medication group interaction term - Adjusted confounders: baseline weight, pre-transplant diabetes, time from transplant to treatment start, metformin use at baseline, insulin use at baseline, and immunosuppression agent. | - Primary endpoint:  changes in HbA1c, body weight, BMI, eGFR, and liver enzymes (AST and ALT). - Secondary endpoint:  the safety (changes in serum immunosuppressants trough levels, graft failure, rejection, malignancy, and death) |

PTDM: post-transplant diabetes mellitus, DM: diabetes mellitus, eGFR: estimated glomerular filtration rate, HbA1c: hemoglobin A1c, FPG: fasting plasma glucose, OGTT: oral glucose tolerance tests, WHR: waist-to-hip ratio, VAT: visceral adipose tissue, BP: blood pressure, UTI: urinary tract infection, T2DM: type 2 diabetes mellitus, ESRD: end-stage renal disease, OHA: oral hypoglycemic agent, BMI: body mass index, SGLT2i: sodium-glucose cotransporter-2 inhibitor, ACE: angiotensin-converting enzyme, ARB: angiotensin 2 receptor blocker, U-ACR: urine albumin creatinine ratio, MI: myocardial infarction, HF: heart failure, CVA: cerebrovascular stroke, LDL: low-density lipoprotein, HDL: high-density lipoprotein, PCI: percutaneous coronary intervention, MACE: major adverse cardiovascular events, SBP: systolic blood pressure, DBP: diastolic blood pressure, ISHLT: international society for heart and lung transplantation, IRS1 and IRS2: insulin receptor substrates 1 and 2, PPAR-γ: peroxisome proliferator-activated receptor-γ, HOMA-IR: homeostatic model assessment for insulin resistance, GLP-1RA: glucagon-like peptide-1 receptor agonist, DPP-4: dipeptidyl peptidase-4, AST: aspartate aminotransferase, ALT: alanine aminotransferase

Table S5. Detailed information regarding the risk of bias assessment according to the Risk of Bias 2 criteria

| **First author, year** | **The risk of bias domain** | **Assessment of risk of bias** | **The reason for judgment** |
| --- | --- | --- | --- |
| Halden, 2019  [24] | Randomization process | Low | Allocation concealed by a randomization list managed by an independent individual.  Baseline characteristics were well balanced between groups |
|  | Deviations from intended interventions | Low | Blinding of both participants and investigators was maintained.  Adherence was monitored |
|  | Missing outcome data | Some concerns | Approximately 10% of patients were excluded during the study period, which could affect the reliability of the results in a small sample size. |
|  | Measurement of the outcome | Low | Outcomes such as HbA1c, body weight, and eGFR were objectively measured using validated methods. Additionally, investigators were blinded to group allocation, reducing the likelihood of measurement bias |
|  | Selection of the reported result | Some concerns | The primary endpoint (change in weighted mean glucose) could not be analyzed due to a technical error, and only secondary endpoints were reported |
|  | Overall | Some concerns |  |

Table S6. Summary of the meta-analysis results for other efficacy outcomes of SGLT2 inhibitors with the control groups

| **Variables** | **No. of**  **studies** | **No. of participants** | | **Estimates** | | **95% CI** | **Heterogeneity** | |
| --- | --- | --- | --- | --- | --- | --- | --- | --- |
|  |  | **SGLT2I** | **Non-**  **SGLT2I** |  |  |  | **I^2^** | **p** |
| *Efficacy* |  |  |  |  |  |  |  |  |
| Body weight (kg) | 6 | 190 | 496 | MD | **-3.00** | **(-4.08, -1.92)** | 0% | 0.96 |
| Serum creatinine (mg/dL) | 5 | 105 | 237 | MD | -0.03 | (-0.12, 0.05) | 0% | 0.79 |

SGLT2I: sodium-glucose cotransporter-2 inhibitors, CI: confidence interval, MD: mean difference

Table S7. Summary of the meta-analysis results for efficacy and safety outcomes within the SGLT2 inhibitor group

| **Variables** | **No. of**  **Studies^*^** | **Estimates** | | **95% CI** | **Heterogeneity** | |
| --- | --- | --- | --- | --- | --- | --- |
|  |  |  |  |  | **I^2^ (%)** | **p** |
| *Efficacy* |  |  |  |  |  |  |
| HbA1c (%) | 11 | MD | **-0.47** | **(-0.71, -0.22)** | 76 | <0.01 |
| Body mass index (kg/m^2^) | 7 | MD | **-0.73** | **(-1.13, -0.33)** | 0 | 0.96 |
| Body weight (kg) | 6 | MD | **-2.12** | **(-3.36, -0.87)** | 0 | 0.86 |
| eGFR (mL/min/1.73m^2^) | 9 | MD | 0.04 | (-0.75, 1.16) | 0 | 0.84 |
| Serum creatinine (mg/dL) | 5 | MD | 0.02 | (-0.08, 0.11) | 0 | 1.00 |
| Dialysis | 2 | Proportion | 0.05 | (0.03, 0.07) | 86 | <0.01 |
| Systolic blood pressure (mmHg) | 5 | MD | -0.72 | (-6.45, 5.01) | 35 | 0.17 |
| Diastolic blood pressure (mmHg) | 5 | MD | -1.69 | (-4.05, 0.66) | 0 | 0.67 |
| Major adverse cardiovascular events | 2 | Proportion | 0.04 | (0.03, 0.05) | 0 | 0.78 |
| Heart failure | 3 | Proportion | 0.05 | (0.00, 0.13) | 98 | <0.01 |
| Myocardial infarction | 2 | Proportion | 0.01 | (0.00, 0.02) | 0 | 0.44 |
| *Safety* |  |  |  |  |  |  |
| Urinary tract infection | 9 | Proportion | 0.11 | (0.07, 0.14) | 66 | <0.01 |
| Genital mycotic infection | 3 | Proportion | 0.01 | (0.00, 0.03) | 0 | 0.69 |
| Acute kidney injury | 3 | Proportion | 0.16 | (0.03, 0.28) | 88 | <0.01 |
| Discontinuation of SGLT2I | 5 | Proportion | 0.08 | (0.05, 0.10) | 32 | 0.21 |
| Graft rejection | 6 | Proportion | 0.15 | (0.01, 0.28) | 99 | <0.01 |
| All-cause hospitalization | 4 | Proportion | 0.32 | (0.10, 0.54) | 99 | <0.01 |
| All-cause mortality | 7 | Proportion | 0.07 | (0.03, 0.10) | 96 | <0.01 |

*Studies reporting zero events were excluded from the pooled proportion analysis.

CI: confidence interval, HbA1c: hemoglobin A1c, SMD: standardized mean difference, eGFR: estimated glomerular filtration rate, SGLT2I: sodium-glucose cotransporter-2 inhibitor


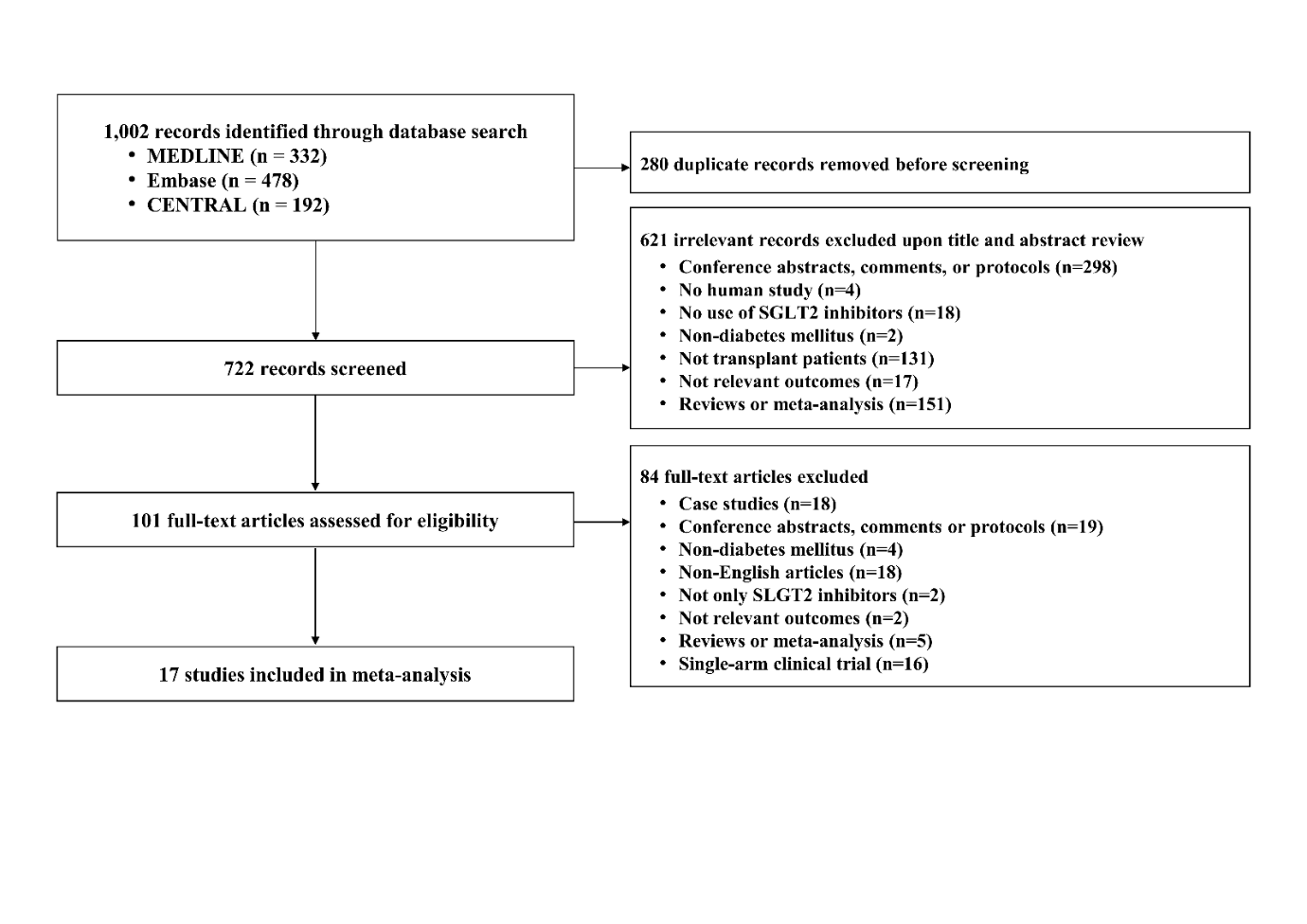


**Fig. S1** Flowchart of the study selection process

SGLT2, sodium-glucose cotransporter-2


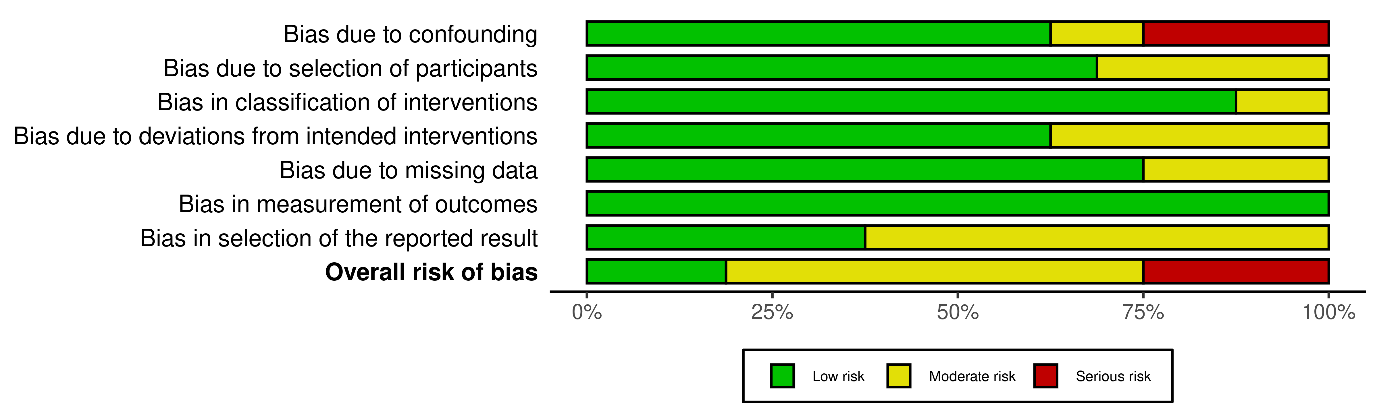


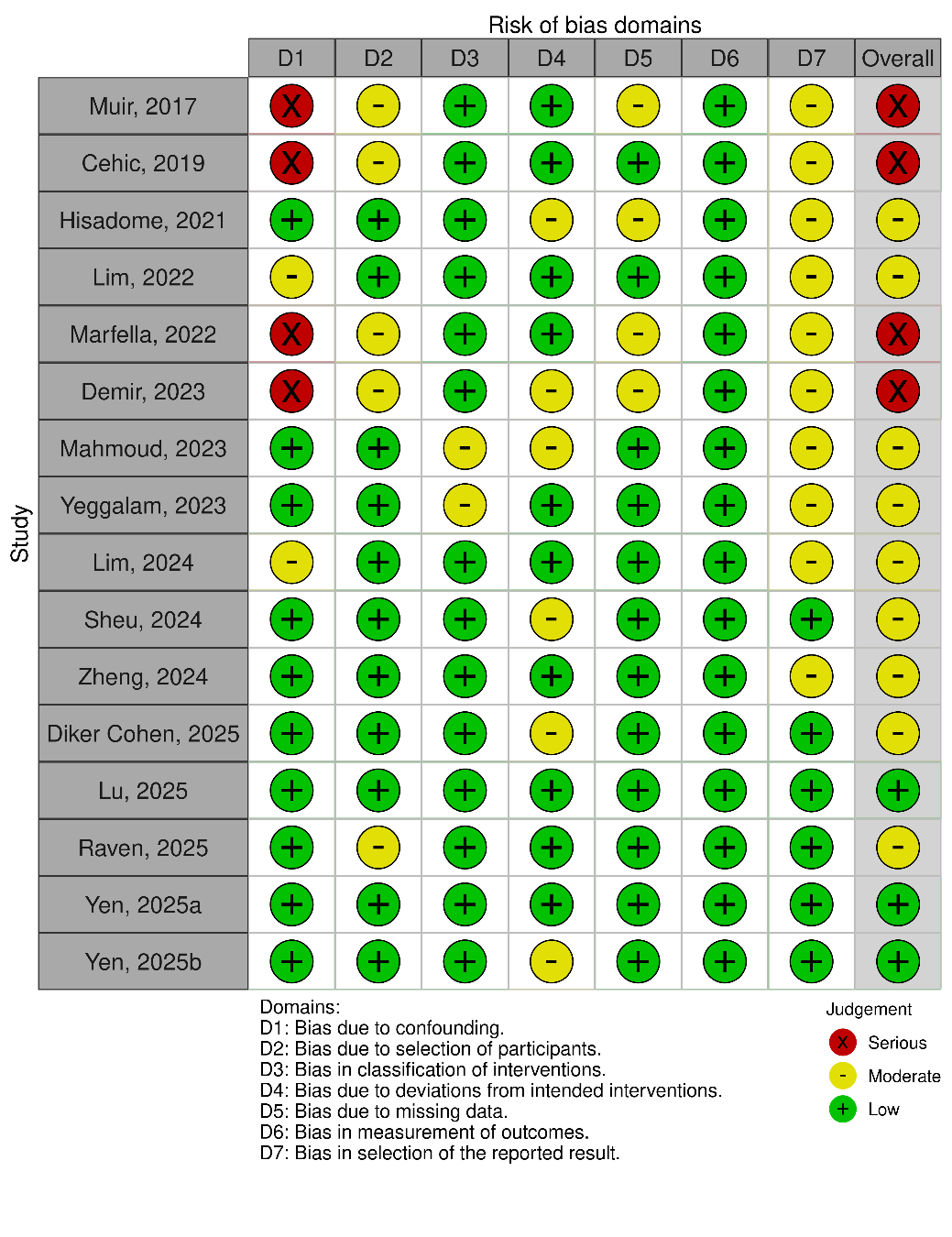


**Fig. S2** Quality assessment of the risk of bias according to the ROBINS-I criteria


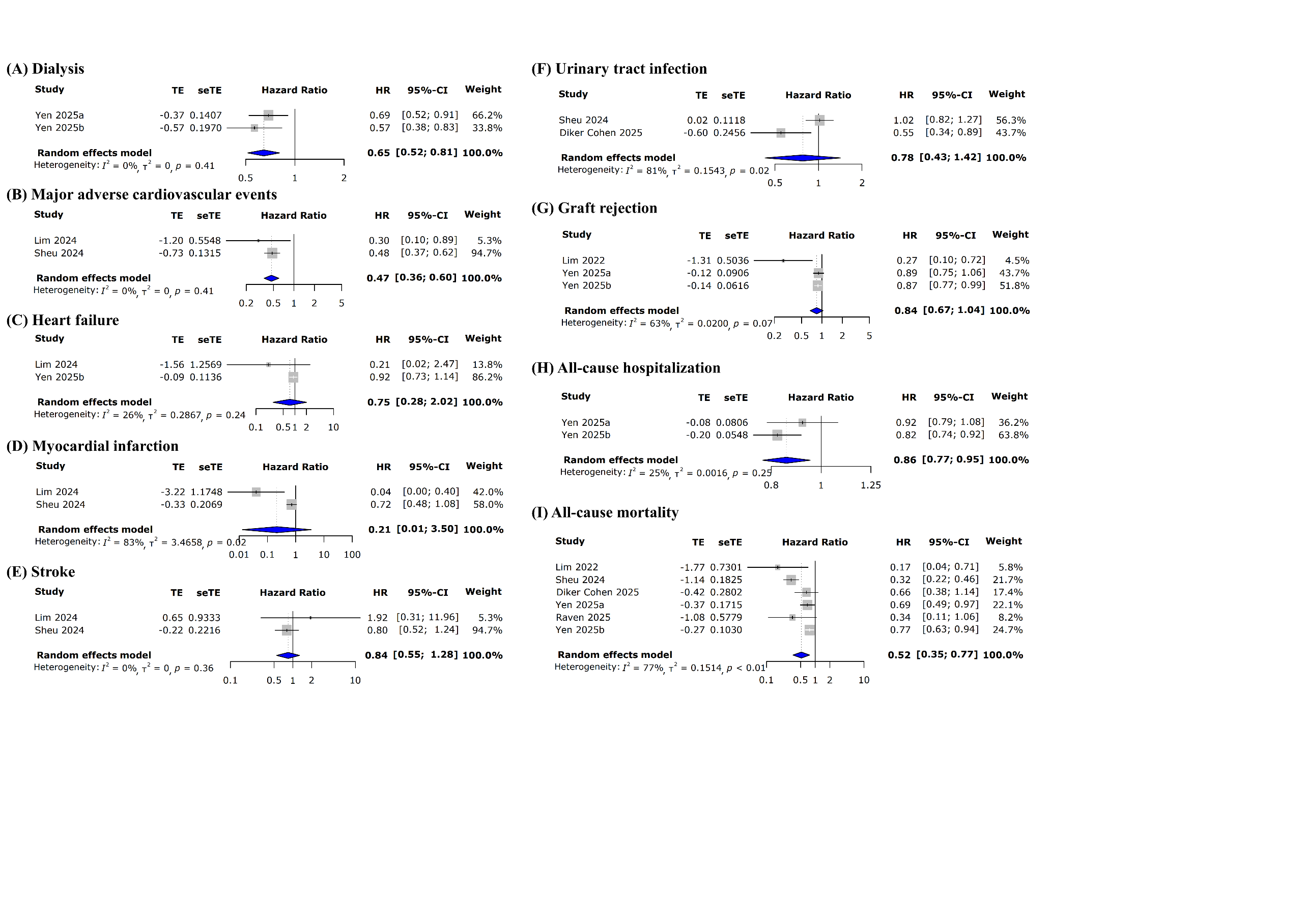


**Fig. S3** Forest plots of hazard ratios

| **(A) HbA1c**  **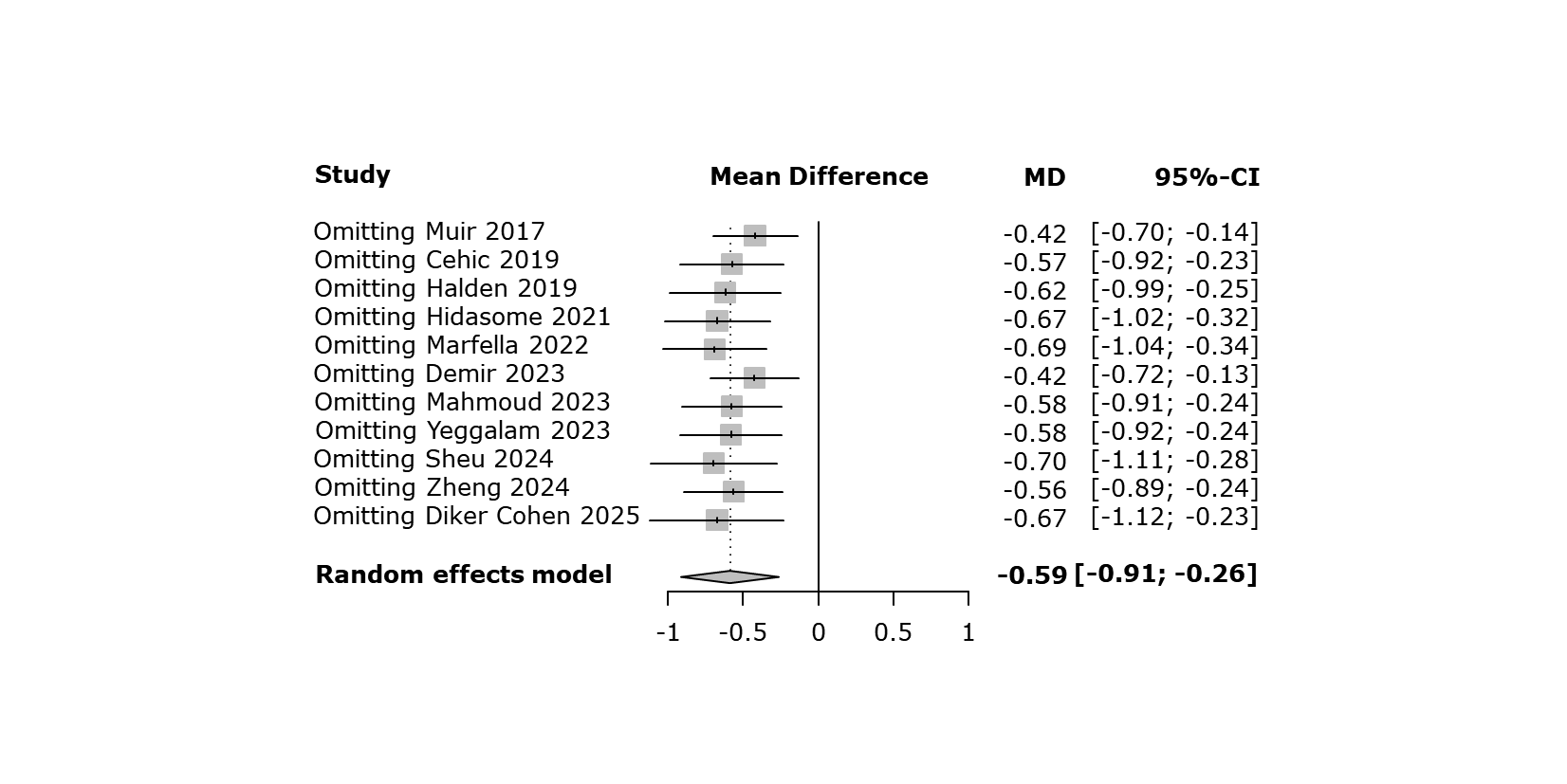**  **(B) Body mass index**  **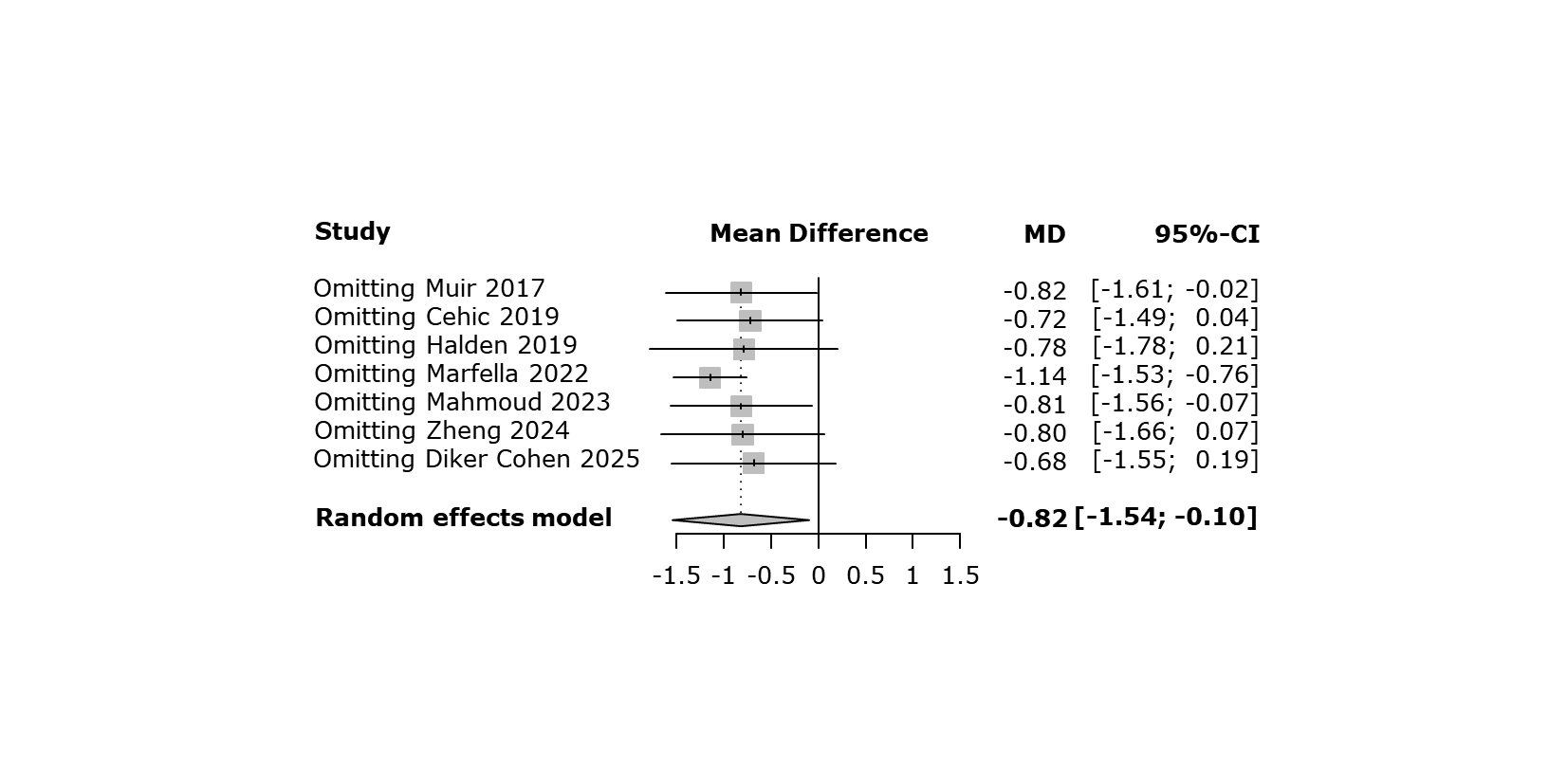**  **(C) Body weight**  **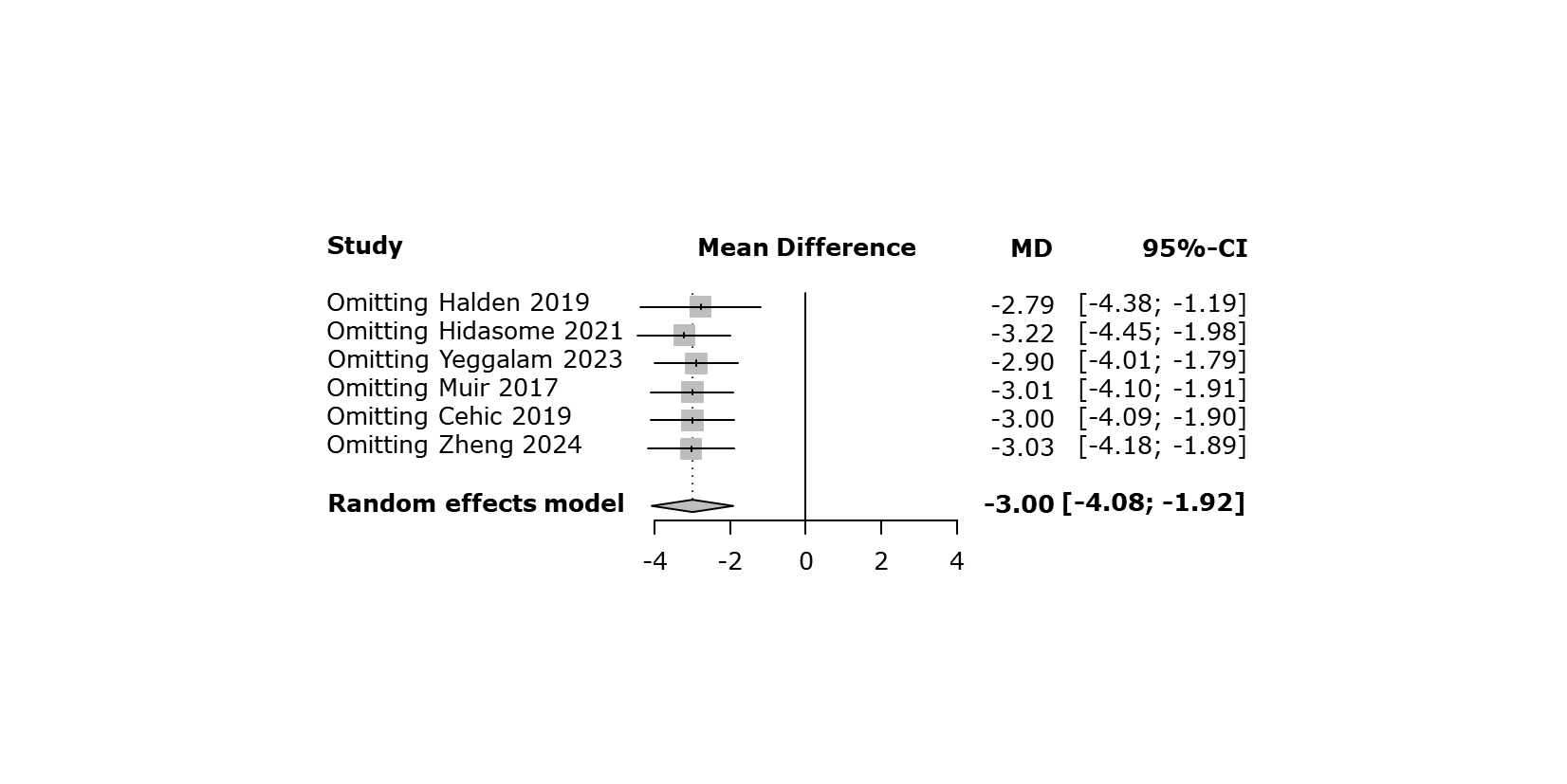**  **(D) eGFR**  **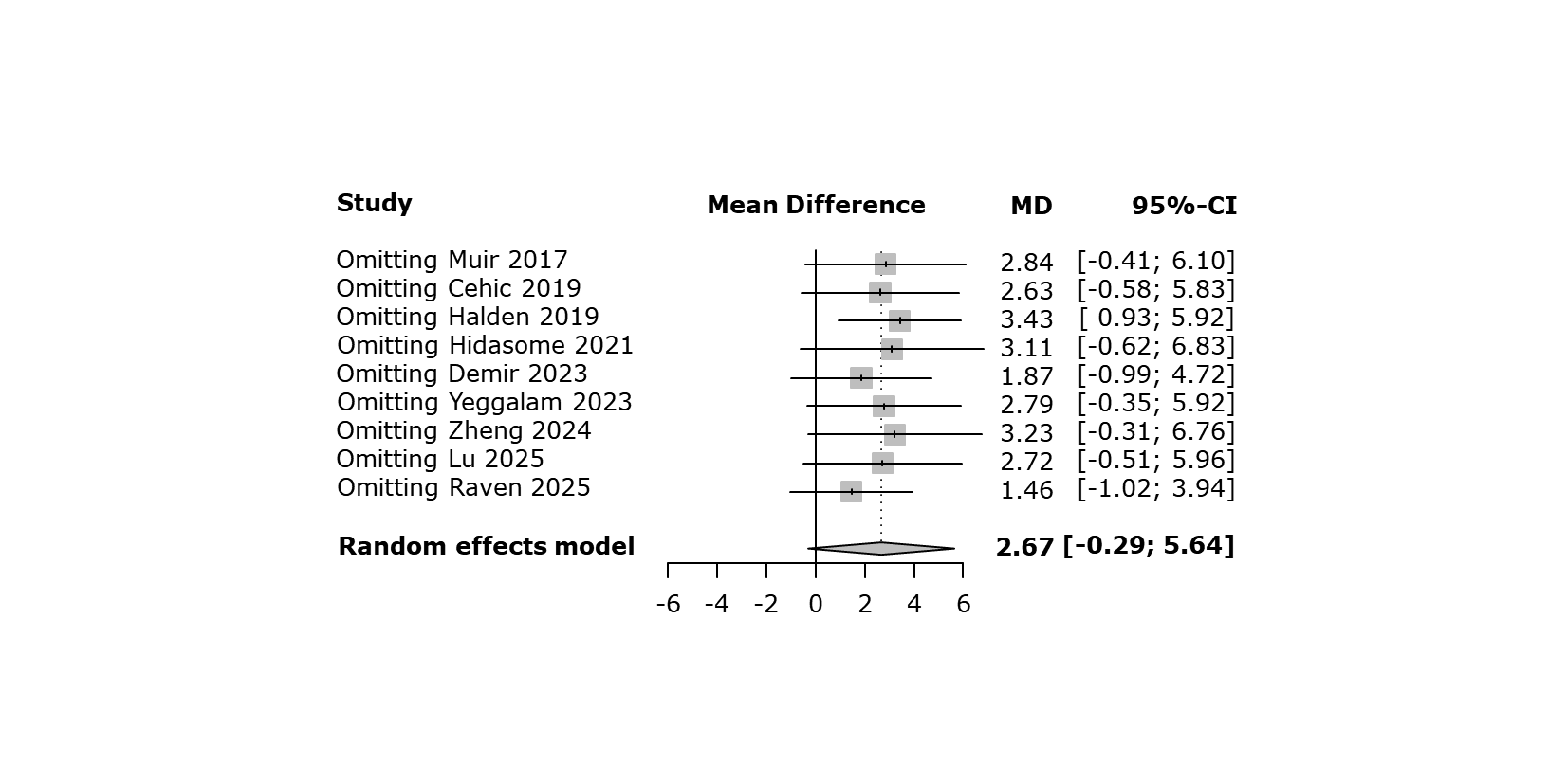**  **(E) Serum creatinine**  **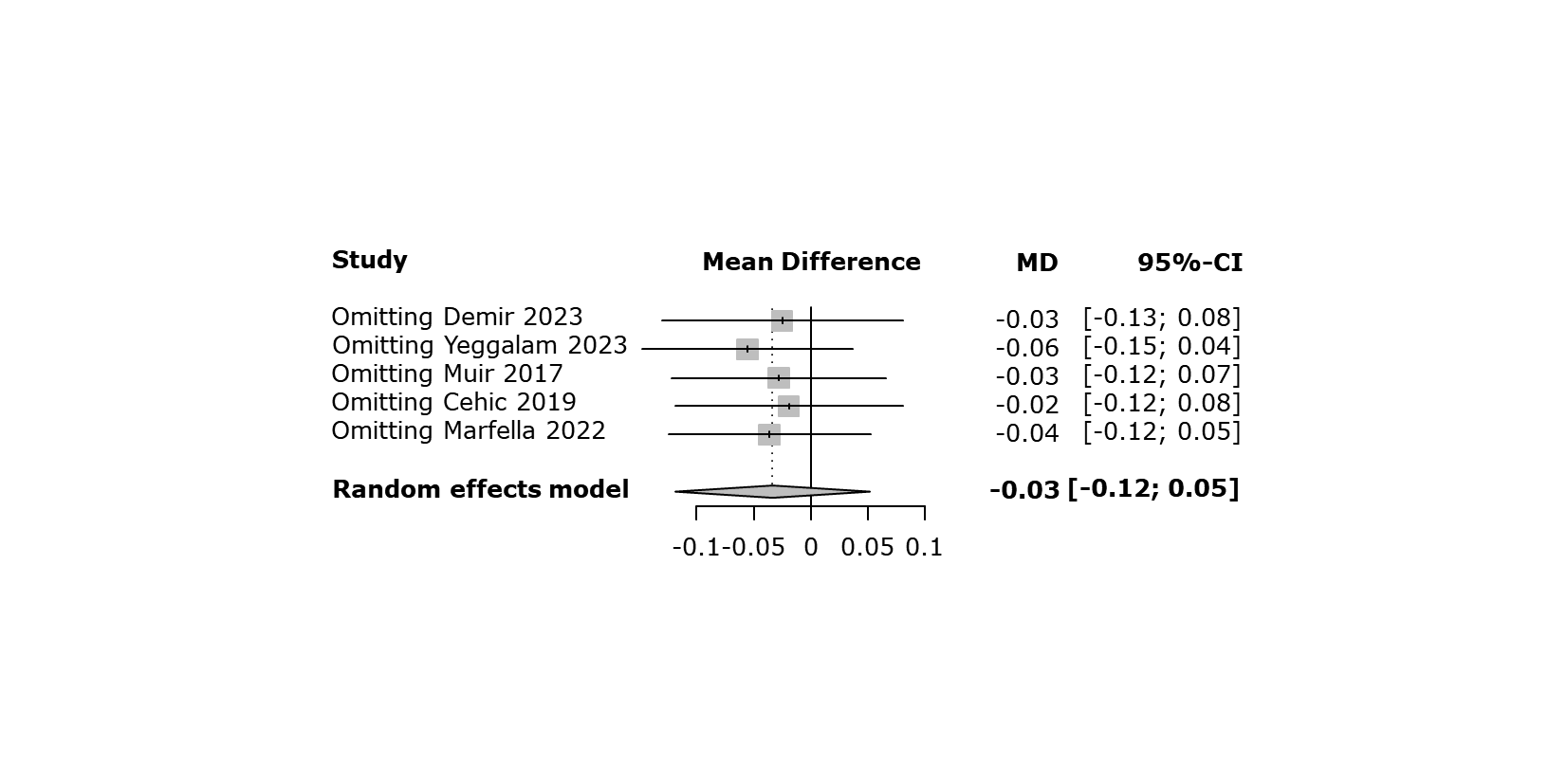** | **(F) Dialysis**  **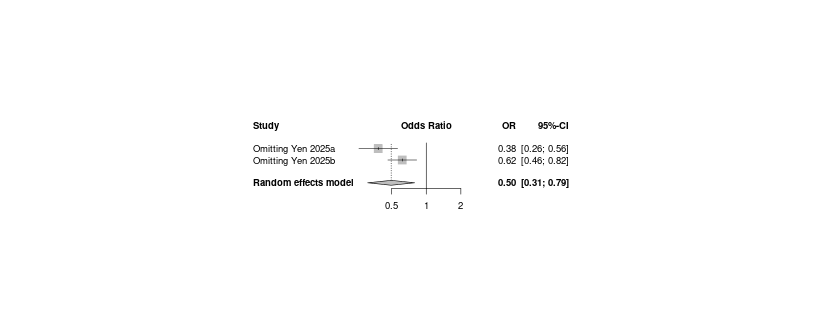**  **(G) Systolic blood pressure**  **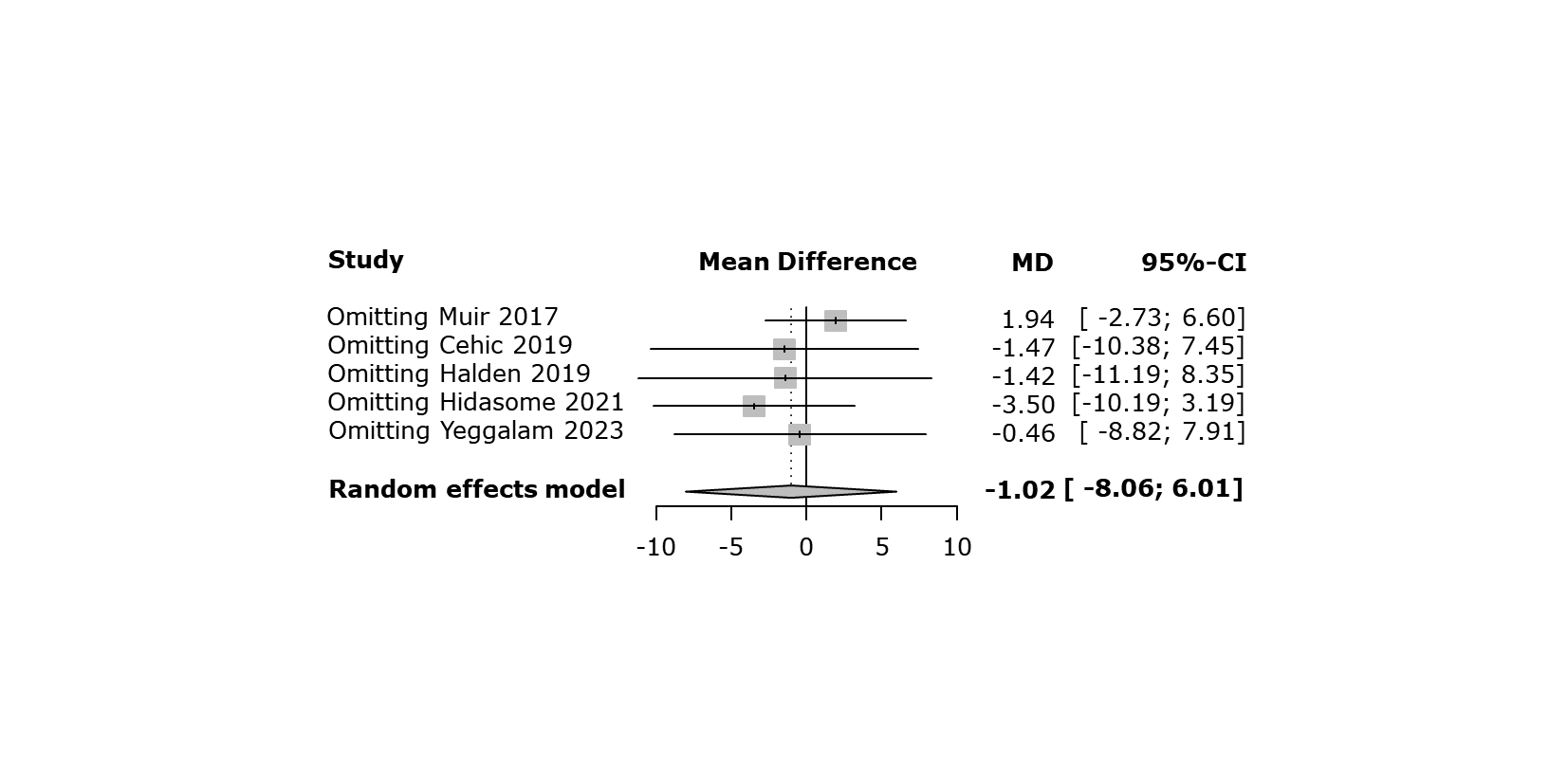**  **(H) Diastolic blood pressure**  **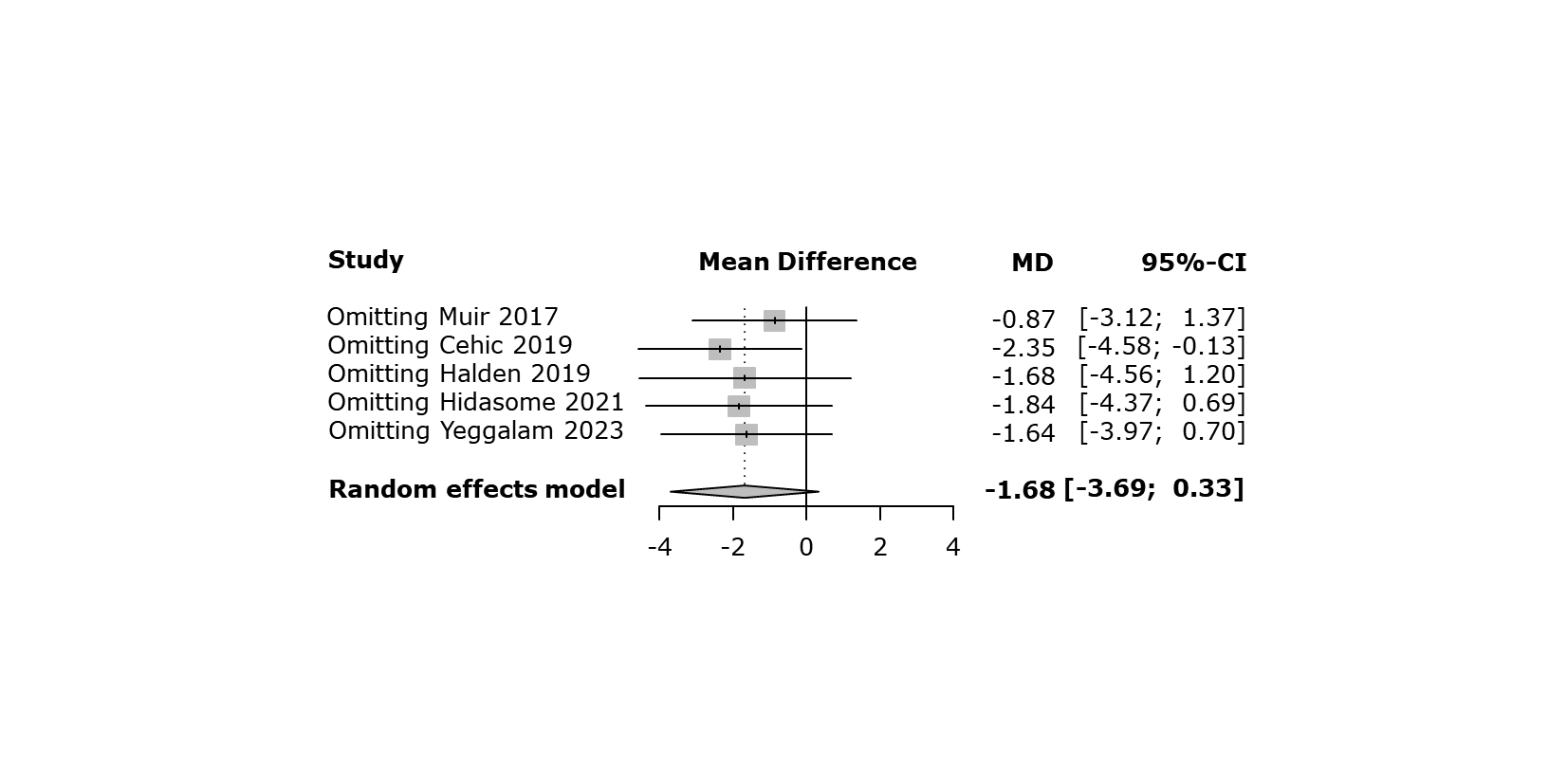**  **(I) MACE**  **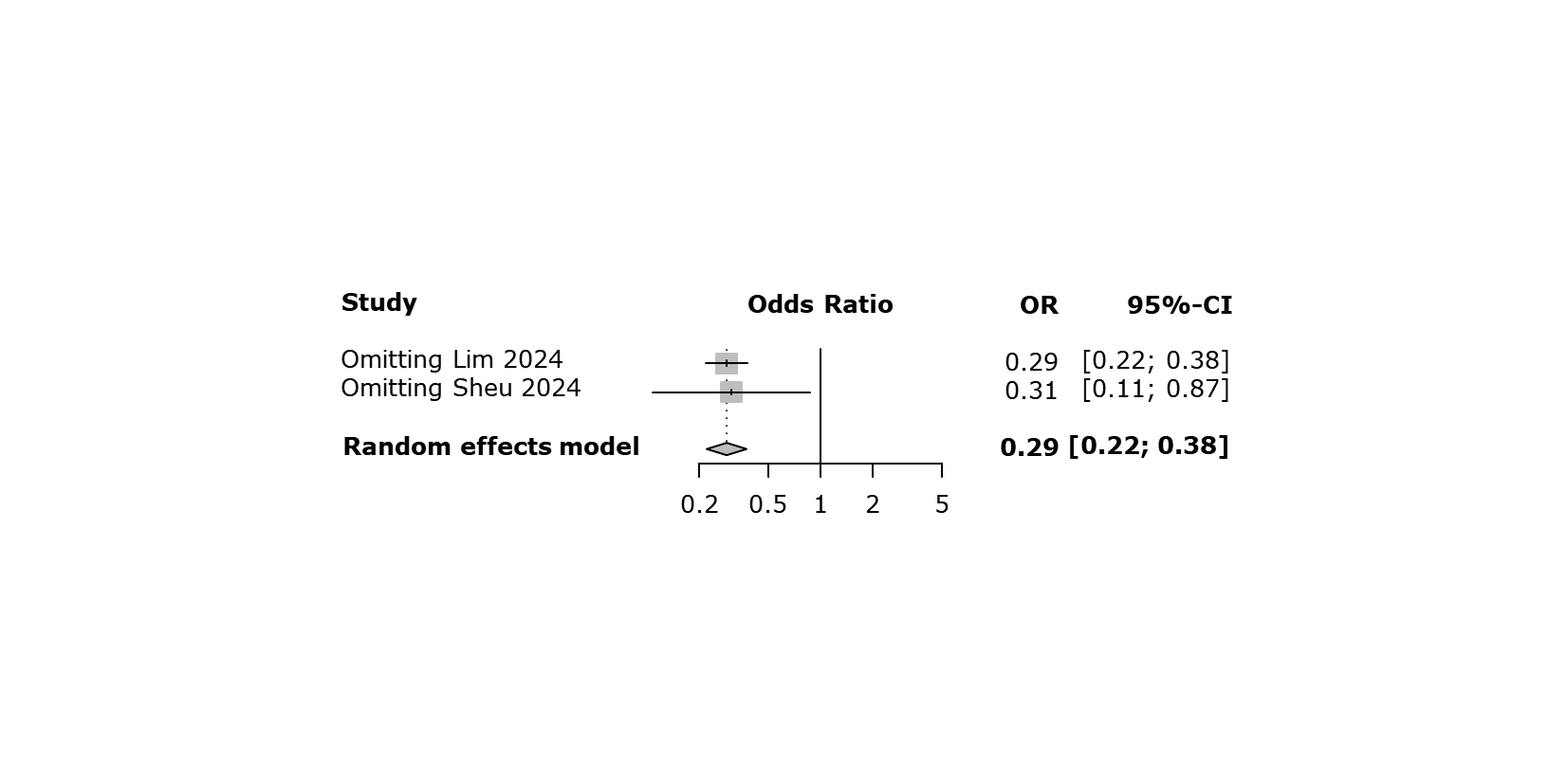**  **(J) Heart failure**  **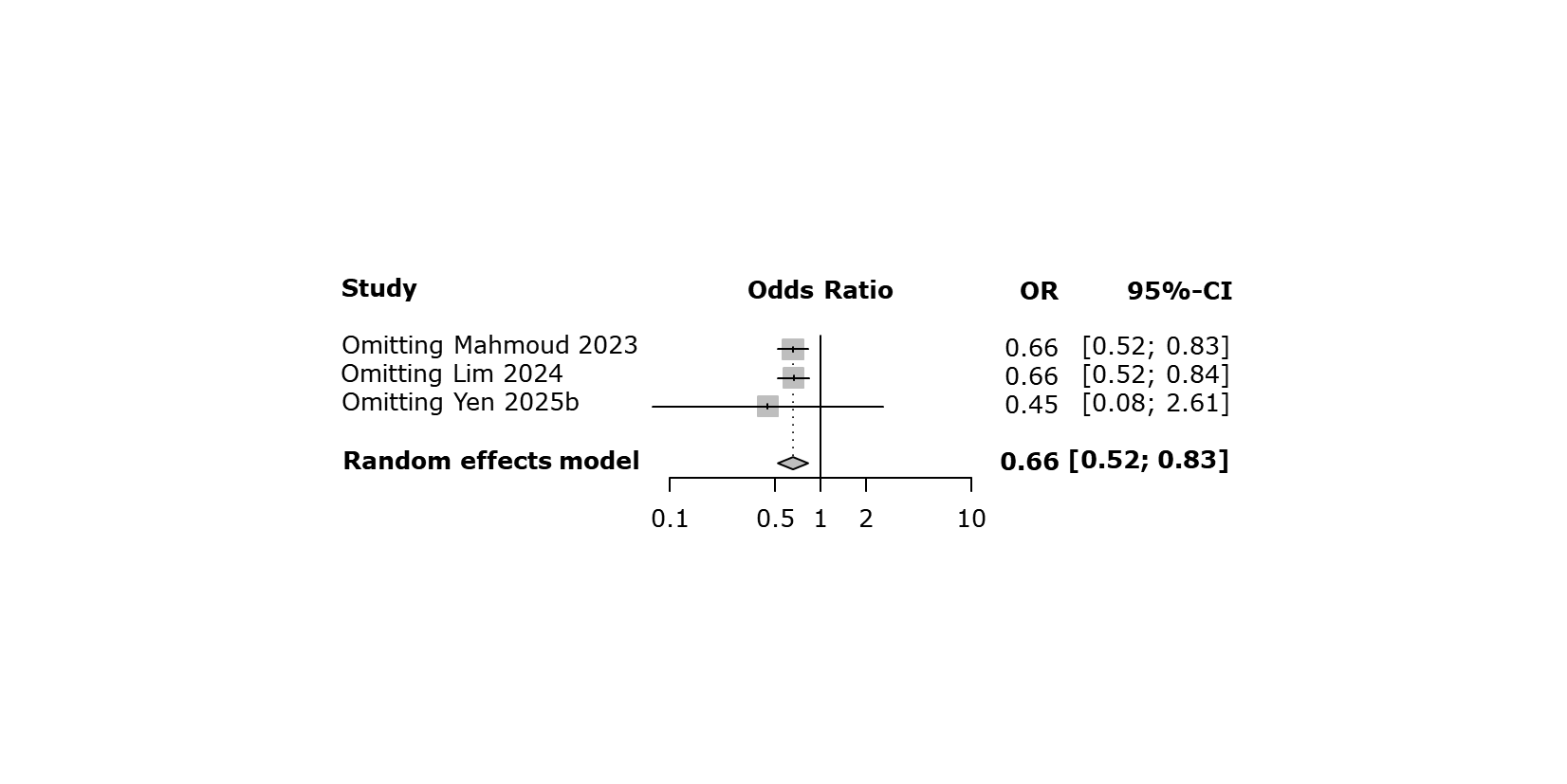**  **(K) Urinary tract infection**  **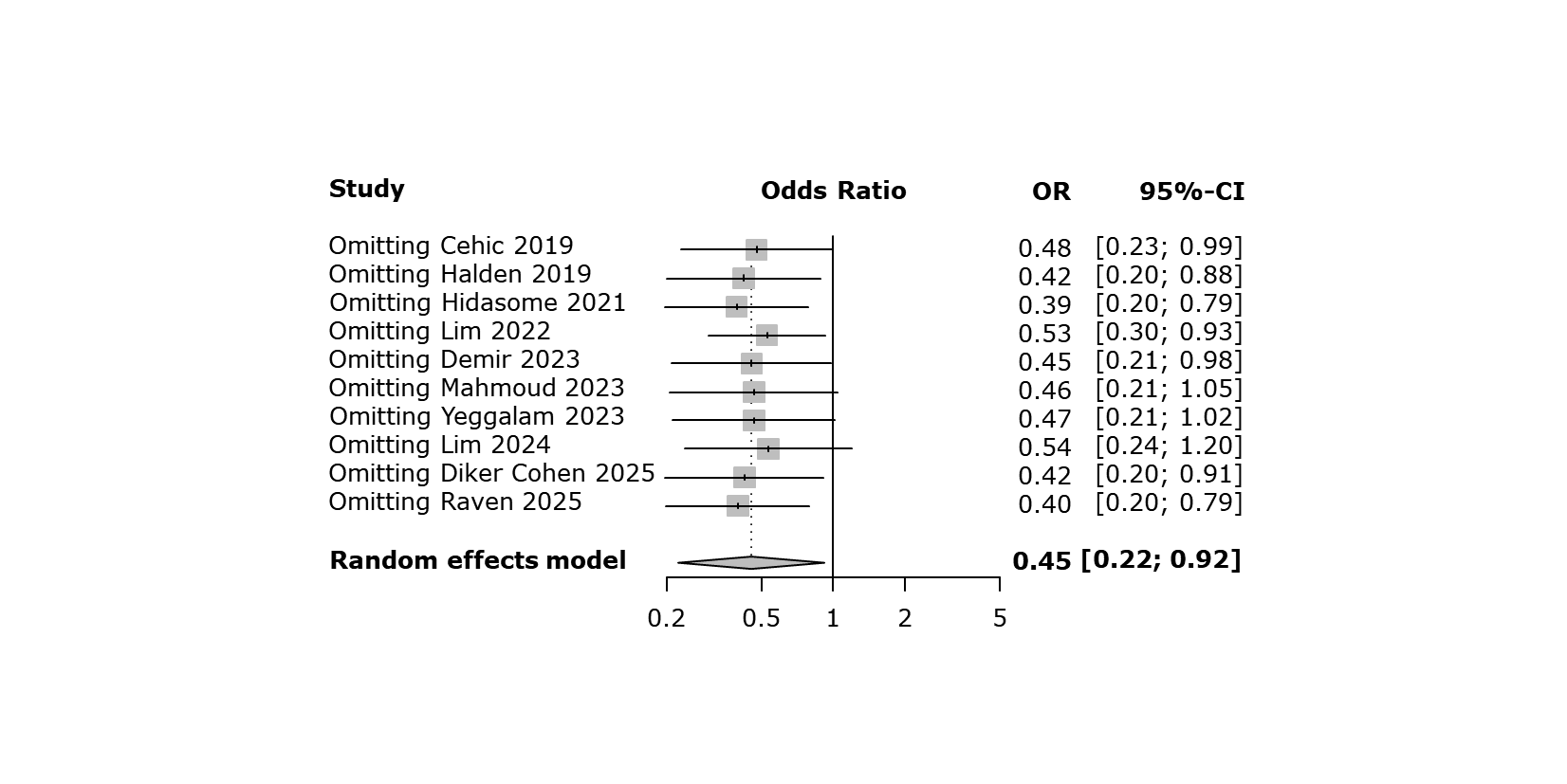**  **(L) Myocardial infarction**  **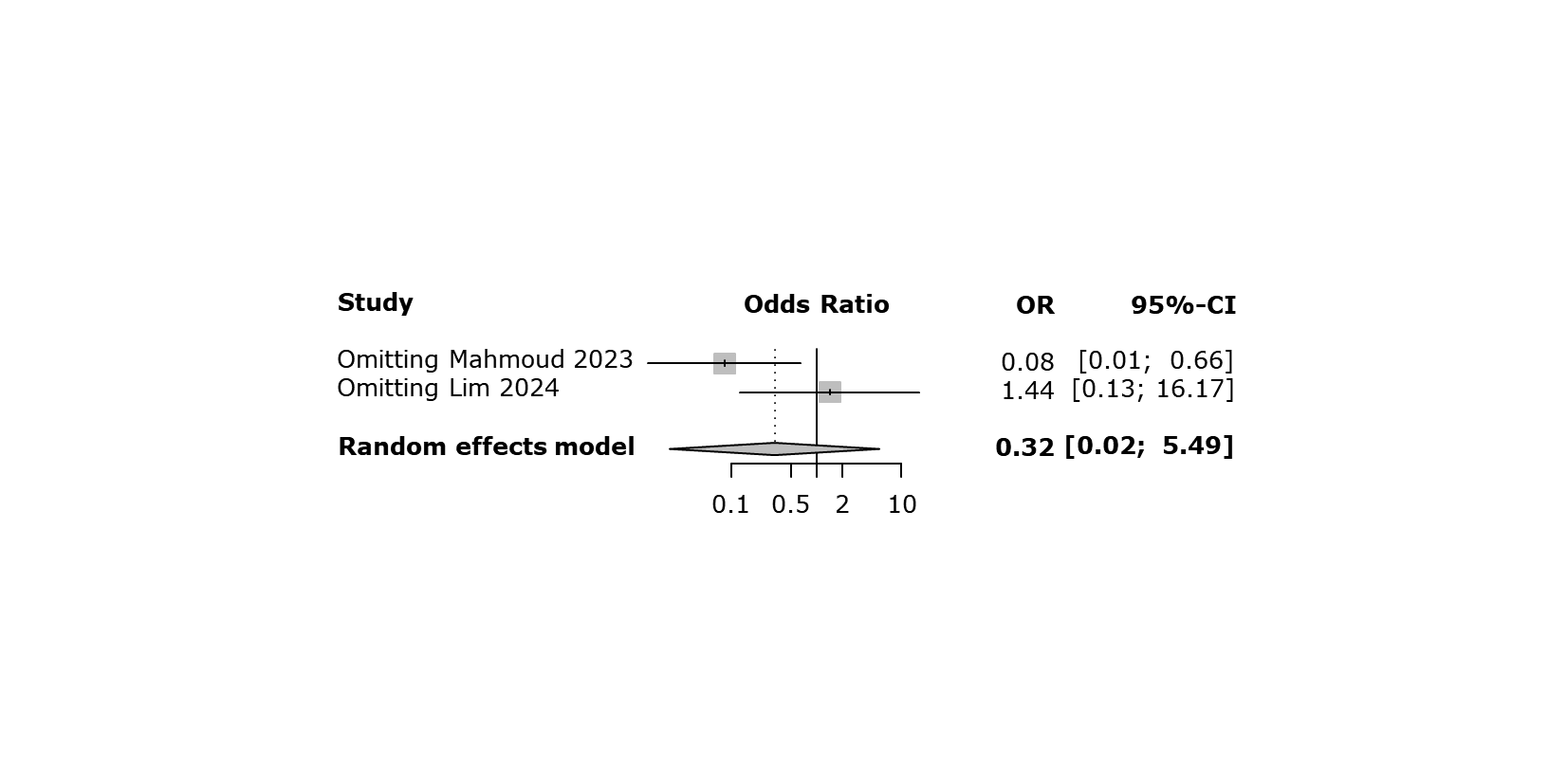** | **(M) Genital mycotic infection**  **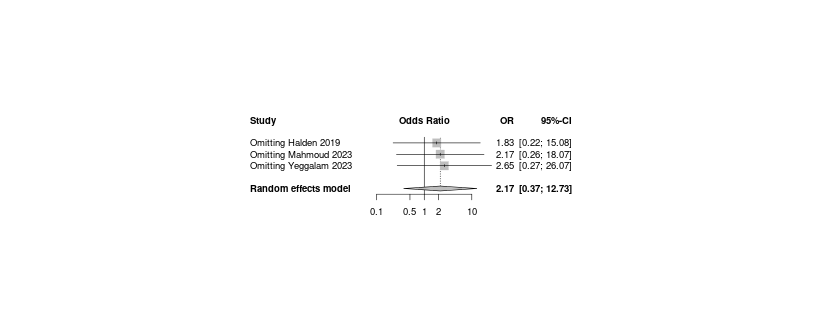**  **(N) Acute kidney injury**  **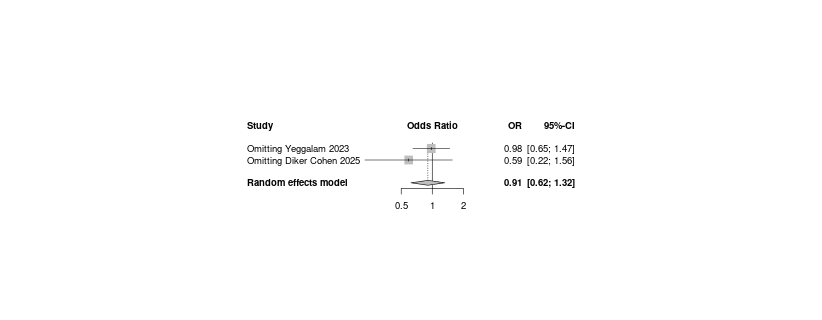**  **(O) Diabetic Ketoacidosis**  **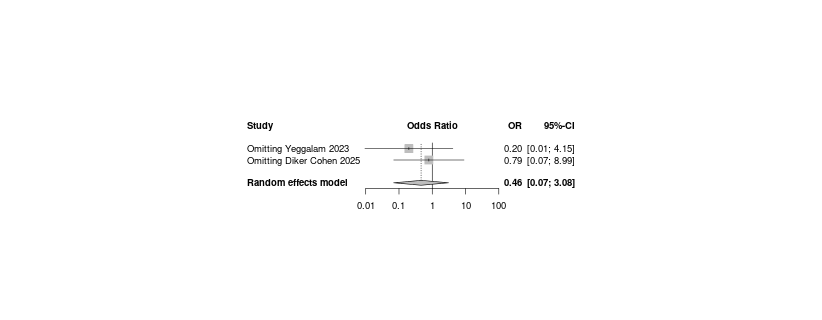**  **(P) Graft rejection**  **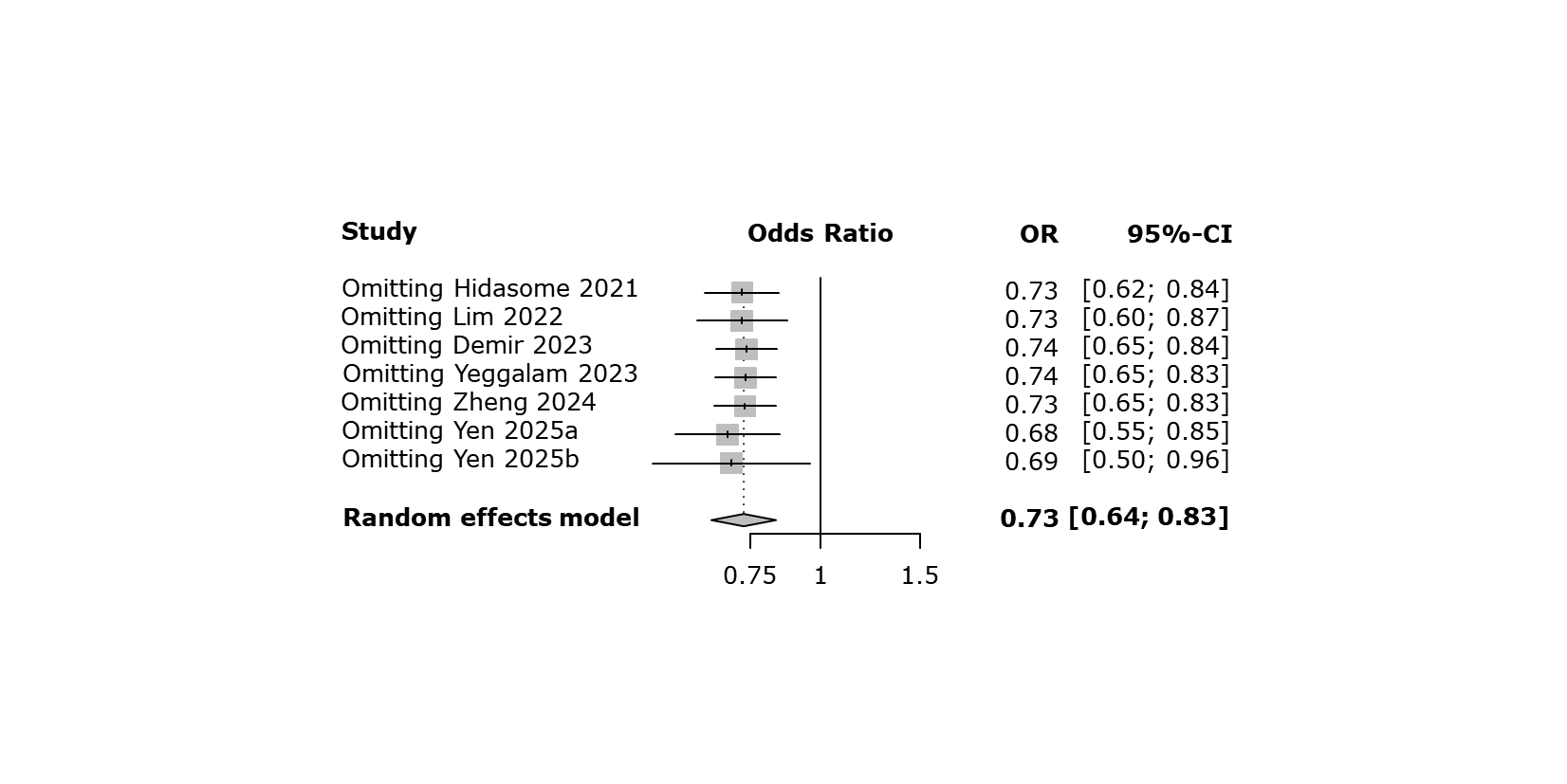**  **(Q) All-cause hospitalization**  **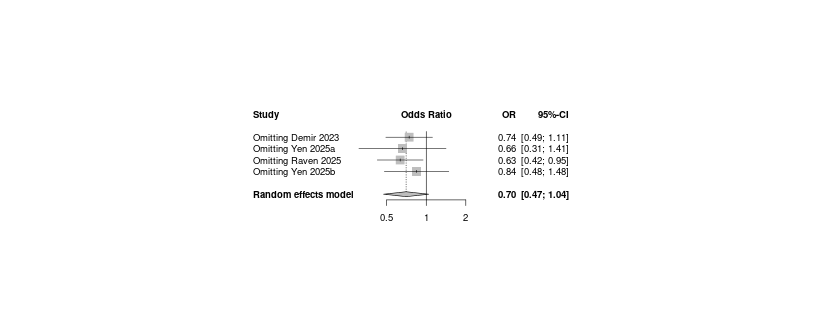**  **(R) All-cause mortality**  **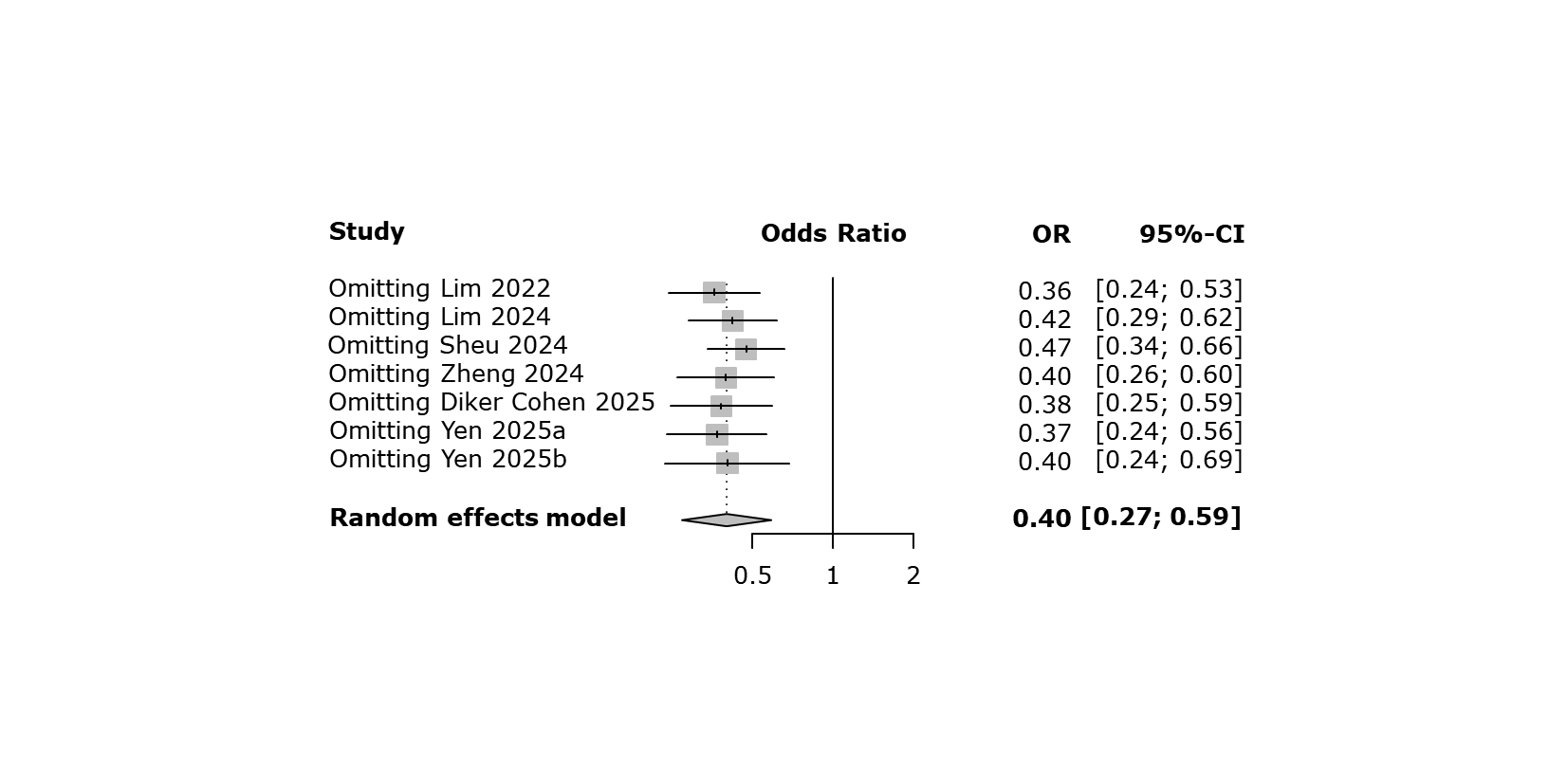** |
| --- | --- | --- |

**Fig. S4** Sensitivity analysis with the leave-one-out method

| (A) HbA1c | (B) Urinary tract infection |
| --- | --- |
| 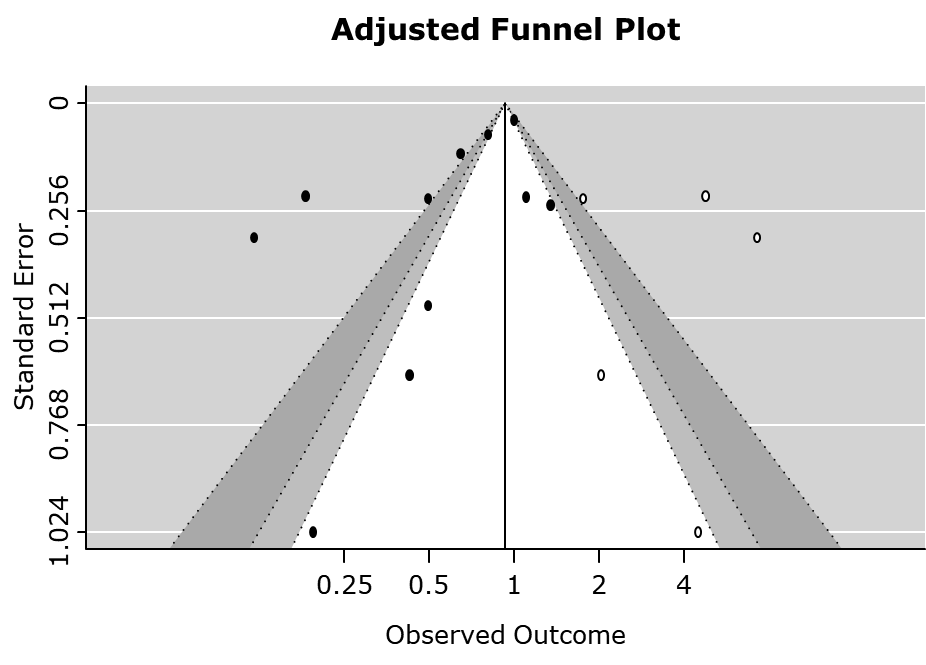 | 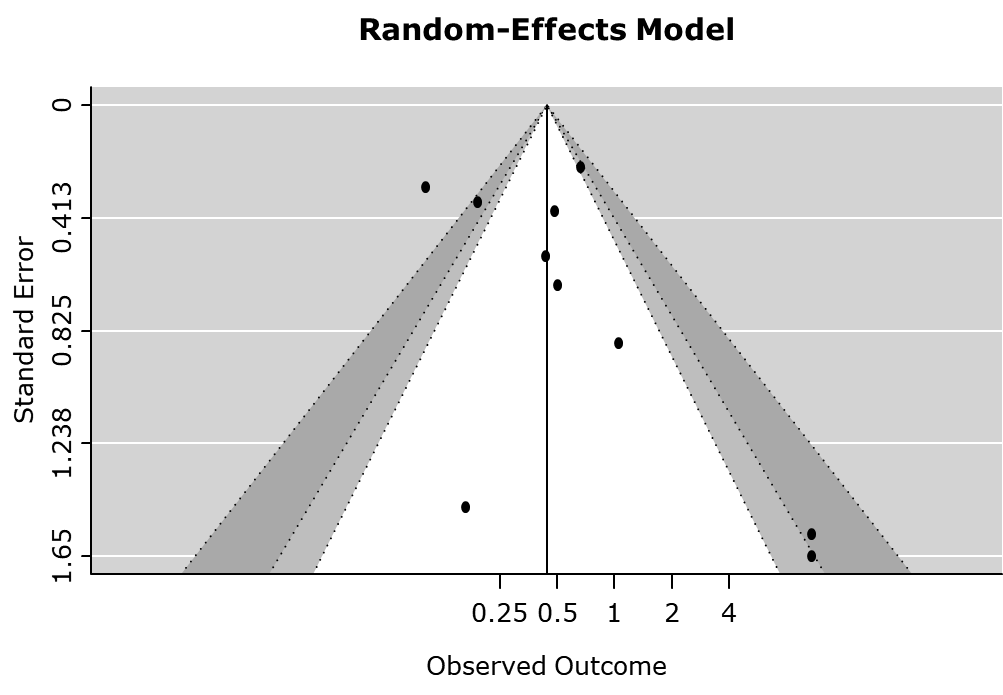 |

**Fig. S5** Funnel plots
